# PockLigGPT: Pocket-Sequence-Conditioned Molecular Generation with GPTs and RL

**DOI:** 10.1021/acs.jcim.6c01980

**Published:** 2026-08-17

**Authors:** Pablo Varas Pardo, Guillermo Marcos-Ayuso, Eugenia Ulzurrun, David Ríos Insua, Nuria E. Campillo

**Affiliations:** † 16722 AItenea Biotech S.L., Madrid 28014, Spain; ‡ 16379 Instituto de Ciencias Matemáticas (ICMAT-CSIC), Madrid 28049, Spain; § Universidad Autónoma de Madrid, Escuela de Doctorado, Madrid 28049, Spain; ∥ Centro de Investigaciones Biológicas Margarita Salas (CIB Margarita Salas−CSIC), Madrid 28040, Spain

## Abstract

*De novo* drug design aims to generate molecules targeting specific protein pockets while retaining chemical plausibility and drug-like properties. Recent 3D structure-based generative methods explicitly model pocket-ligand geometry, but this does not always translate into chemically realistic or practically usable candidate molecules. Molecular language models provide a complementary sequence-based alternative. However, it remains unclear whether sequence-based pocket information can effectively guide ligand generation, whether multistage training improves pocket-specific generation, and whether docking-guided reinforcement learning can be integrated into a practical generation pipeline. We introduce PockLigGPT, a GPT-based framework for pocket-sequence-conditioned molecular generation. Rather than producing fixed 3D coordinates, PockLigGPT formulates ligand design as a sequence-generation problem conditioned on the amino acid composition of the protein pocket. The model is trained in four stages: large-scale chemical pretraining based on ZINC20; bioactivity-oriented adaptation based on ChEMBL; pocket-sequence-conditioned fine-tuning using binding-pocket amino acid sequences paired with ligands; and, finally, pocket-specific docking-guided reinforcement learning using AutoDock Vina-based rewards. PockLigGPT achieves competitive docking-oriented performance under a standardized evaluation protocol while maintaining chemical plausibility, favorable physicochemical profiles, and Lipinski-based drug-likeness. Docking studies on Alzheimer’s disease-associated targets and token-level analyses further support the utility of PockLigGPT for *de novo* drug design.

## Graphical abstract

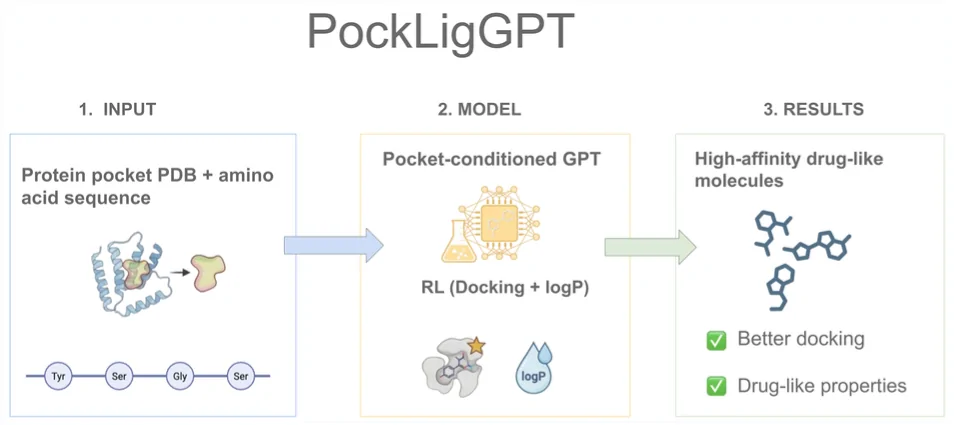

## Introduction

Designing drug-like molecules that selectively bind specific protein pockets remains a major challenge in early stage drug discovery. This difficulty is largely driven by the vast size of the chemical space, estimated to contain between 10^30^ and 10^60^ compounds.^1,2^ Machine learning can help address this challenge by generating molecules with favorable predicted binding affinity and drug-like physicochemical profiles.^3–6^

Many current *de novo* drug design methods employ *3D structure-based models*. These methods generate molecules directly inside a protein pocket by predicting atom types and 3D coordinates. Examples include Pocket2Mol,^7^ AR,^8^ TargetDiff,^9^ DiffSBDD,^10^ and Lingo3DMol.^11^ These approaches explicitly use the binding-site geometry and can generate molecules conditioned on a target pocket. However, their practical use remains challenging: the generated molecules may still display unrealistic geometries, limited drug-likeness, or suboptimal physicochemical profiles, even when their predicted 3D placement appears compatible with the binding pocket.^12,13^ In addition, the generated poses often require redocking or pose refinement before evaluation.^14^ This additional refinement can substantially alter the initially generated coordinates, reducing the practical relevance of the original pose prediction.^15,16^

*Molecular language models* provide a complementary strategy. Instead of directly generating 3D coordinates, they represent molecules as text, typically using either the Simplified Molecular Input Line Entry System (SMILES)^17^ or SELF-referencing Embedded Strings (SELFIES).^18^ This enables the use of autoregressive sequence models that learn chemical syntax from large molecular databases and generate molecular strings token by token.^19,20^ This paradigm is attractive because modern language models can be adapted through pretraining, prompting, fine-tuning, and reinforcement learning (RL).^21–23^

Recent studies have explored related directions. DrugGPT^24^ introduces protein-conditioned molecular generation using textual protein information, but relies on full protein sequences and does not include docking-guided RL. BindGPT^25^ explores a structure-aware language-modeling strategy by combining molecular representations with 3D structural information in a multistage framework. These studies suggest that conditioned molecular generation is a promising direction.

However, several questions remain open when trying to transfer this paradigm to pocket-conditioned molecular generation. First, it remains unclear whether sequence-based pocket information can effectively guide ligand generation; second, the benefits of multistage training for adaptation to specific protein pockets are not yet well understood; and third, it remains important to test whether such a pipeline can improve binding-oriented metrics while preserving chemical plausibility.

Motivated by these limitations, we introduce PockLigGPT, a pocket-sequence-conditioned Generative Pretrained Transformer (GPT) framework to generate drug-like ligands. Our system does not directly generate fixed 3D ligand coordinates. Instead, it follows a pocket-sequence-informed language-modeling strategy such that the amino acid sequence of the binding pocket is used as input context during molecular generation. A generative policy is subsequently refined using docking feedback.

Our pipeline consists of four stages. First, we pretrain a GPT-2 Medium model^26^ on ZINC20 ^27^ to learn a broad chemical prior. Second, we adapt the model on ChEMBL^28^ to bias generation toward bioactive and pharmacologically relevant molecules. Third, we introduce pocket-sequence information through fine-tuning over pocket–ligand pairs from the CrossDocked^29^ and the Jglaser binding-affinity data sets.^30–33^ Finally, we apply Proximal Policy Optimization (PPO) using a reward that combines AutoDock Vina docking scores^34,35^ with physicochemical regularization.

Thus, our main contributions in this paper are

- Propose PockLigGPT, a pocket-sequence-conditioned molecular language model for *de novo* ligand generation.
- Design a multistage training strategy combining large-scale chemical pretraining, ChEMBL-based bioactivity adaptation, pocket-sequence-conditioned fine-tuning, and docking-guided RL.
- Show that pocket-sequence conditioning and docking-guided RL improve docking-oriented performance while preserving chemical plausibility, favorable physicochemical profiles, and Lipinski-based drug-likeness.
- Evaluate top-ranked generated candidates on Alzheimer’s disease-associated targets, including Dual-Specificity Tyrosine-Phosphorylation-Regulated Kinase 1A (DYRK1A),^36^ Butyrylcholinesterase (BuChE),^37^ and Beta-secretase 1 (BACE-1).^38^
- Analyze how docking-guided RL reshapes molecular generation by examining token-level chemical changes before and after optimization.

## Related Work and Motivation

### Text-Based Molecular Generation

Text-based molecular generation represents molecules as sequences, typically using SMILES or SELFIES. This formulation enables the use of sequence models originally developed for natural language processing (NLP), including recurrent neural networks^39^ and the recent transformer architectures.^21^

Recent work has increasingly used large-scale molecular pretraining to learn broad chemical priors before task-specific adaptation. Decoder-only models such as MolGPT^40^ and DrugGPT^24^ formulate molecular generation as autoregressive modeling, whereas pretrained chemical language models have been used for activity-focused *de novo* design and pocket-specific ligand prioritization.^20^ More broadly, large language models (LLMs) and domain-specific language models have been discussed as emerging tools within drug discovery.^19^

Beyond standard SMILES generation, recent approaches have explored richer token-based and conditional formulations. Token-Mol^41^ and 3DSMILES-GPT^42^ illustrate the growing interest in token-based molecular representations that incorporate structural information and can be adapted through task-specific fine-tuning. Encoder-decoder architectures have also been explored for scaffold-conditioned generation, as in T5MolGe,^43^ where molecular scaffolds are encoded to guide SMILES generation and transfer learning is used for EGFR inhibitor-like molecule generation.

### Property Optimization through Reinforcement Learning

RL has been used to fine-tune molecular generators in pursuit of desired properties using reward functions. REINVENT^44–46^ established RL as a practical strategy for optimizing molecular generators with recurrent and transformer-based architectures. Transformer-based SMILES generators have also been combined with policy-gradient RL, as in Taiga,^47^ where a pretrained SMILES language model is fine-tuned toward molecular properties.

The choice of the involved RL algorithm is also relevant. Comparative studies of SMILES-based molecular optimization have shown that policy optimization and replay strategies influence both molecular quality and diversity.^48^ In any case, a key limitation of RL-based molecular generation systems is mode collapse, when the adopted model exploits narrow high-reward regions and generates structurally similar molecules. Diversity-aware reward shaping, including scaffold-based penalties and intrinsic rewards, has been proposed to mitigate this issue.^49^ Mol-AIR^50^ further combines history-based and learning-based intrinsic rewards, including random network distillation, to improve exploration in goal-directed molecular generation. These studies highlight the central role of reward engineering in balancing optimization and molecular diversity.

Docking-based rewards are especially attractive because they provide structure-based feedback. However, integrating docking into an RL-driven molecular generation pipeline is technically challenging. Docking requires ligand preparation, conformer generation, receptor preparation, binding-site definition, docking execution, and postprocessing.^51^ Docking scores also depend strongly on the docking engine, receptor preparation, ligand protonation, and search-space definition.^52^

Historically, AutoDock- and Vina-based workflows have relied on the MGLTools software suite for ligand and receptor preparation, particularly for conversion into the PDBQT format, which extends PDB files with partial atomic charges and AutoDock atom types.^53,54^ More recent tools such as Meeko^55^ improve these workflows by providing a Python-based parametrization framework based on RDKit chemical perception. Such tools are important for RL pipelines, where thousands of generated molecules must be prepared, docked, scored, and filtered.

Several molecular generators have directly used docking as part of the reward. MolRL-MGPT^56^ included docking in a multiobjective reward for SARS-CoV-2 inhibitor designs. MolOrgGPT^57^ pretrained decoder-only GPT models on ZINC20, then fine-tuned them with PPO using docking-based rewards against Alzheimer’s disease-related targets. BindGPT also uses docking-guided RL in a structure-aware framework that combines molecular strings with 3D conformational information. These works show that docking-guided RL is promising, but computationally expensive and sensitive to preprocessing choices.

### Pocket Conditioning in Molecular Language Models

Pocket-conditioned molecular generation aims to bias molecular generation toward molecules compatible with a specific binding site. Recent molecular language models have explored different ways of converting pocket-ligand geometry into sequential inputs. Lingo3DMol^11^ represents pockets as sequences of residue- and atom-level nodes enriched with discretized 3D coordinates and chemical labels, using interaction-relevant regions as anchors for generation. TamGen^58^ adopts an encoder–decoder formulation in which a 3D-aware pocket sequence guides molecular string generation through cross-attention. 3DSMILES-GPT^42^ further extends token-based generation by representing molecular strings, atomic coordinates, and pocket surface information as tokens, with a detachable pocket encoder for pocket feature extraction. BindGPT^25^ represents a structure-aware language-modeling strategy that encodes molecular graphs, 3D conformations, and protein pockets as textual tokens, combining 3D molecular pretraining, pocket–ligand fine-tuning, and docking-guided optimization.

A related line of work uses target information without explicit 3D structures. For example, protein-sequence-conditioned generation has been formulated as translation from amino acid sequences to SMILES strings,^59^ and further improved through pretrained biochemical language models for protein-to-molecule generation.^60^ GPT-based frameworks have also conditioned molecular generation on protein sequence information.^24^ More recent target-aware chemical language models, such as Chem42,^61^ encode full protein sequences with protein language models and inject the resulting embeddings into chemical language models through cross-attention. Related multimodal biochemical language models have further combined protein sequence embeddings with potency-value conditioning to generate compounds with desired activity levels.^62^

### Motivation

Our proposal, PockLigGPT, is motivated by a change in the training paradigm of modern language models. In NLP, models are rarely optimized for a final task from scratch. Instead, they first learn broad priors through large-scale pretraining that are subsequently adapted to a desired task through conditioning, fine-tuning, or RL.

A similar strategy is natural for molecular generation. However, learning chemical syntax from large molecular databases is quite different from learning the bioactive chemical space, and both approaches differ from generating ligands for a specific binding pocket. Moreover, molecular suitability is inherently target-dependent: a molecule that is favorable for one pocket may be suboptimal for another. Direct optimization with a final docking reward can provide a sparse, noisy, and computationally expensive signal. A staged pipeline therefore provides a more structured path: first learning valid chemistry; then adapting toward bioactive molecules; next introducing pocket-dependent information through pocket–ligand fine-tuning; and, finally specializing the generator for a selected pocket through pocket-specific docking-guided RL.

Although multistage training is common in language modeling, its role in pocket-conditioned molecular generation remains less systematically characterized. PockLigGPT explores this idea in a lightweight SELFIES-based framework, where pocket information guides molecular string generation without requiring the model to directly generate fixed 3D ligand coordinates.

A second motivation concerns how target information is provided to the generator. Protein-sequence-conditioned models have shown that biological context can be incorporated into autoregressive molecular generation. However, full protein sequences contain substantial information unrelated to ligand binding, whereas ligand design is primarily driven by the binding-site environment, as Figure 1 shows. PockLigGPT therefore promotes pocket-level conditioning as a biologically motivated and computationally efficient alternative to full-sequence conditioning.

**Figure 1:**
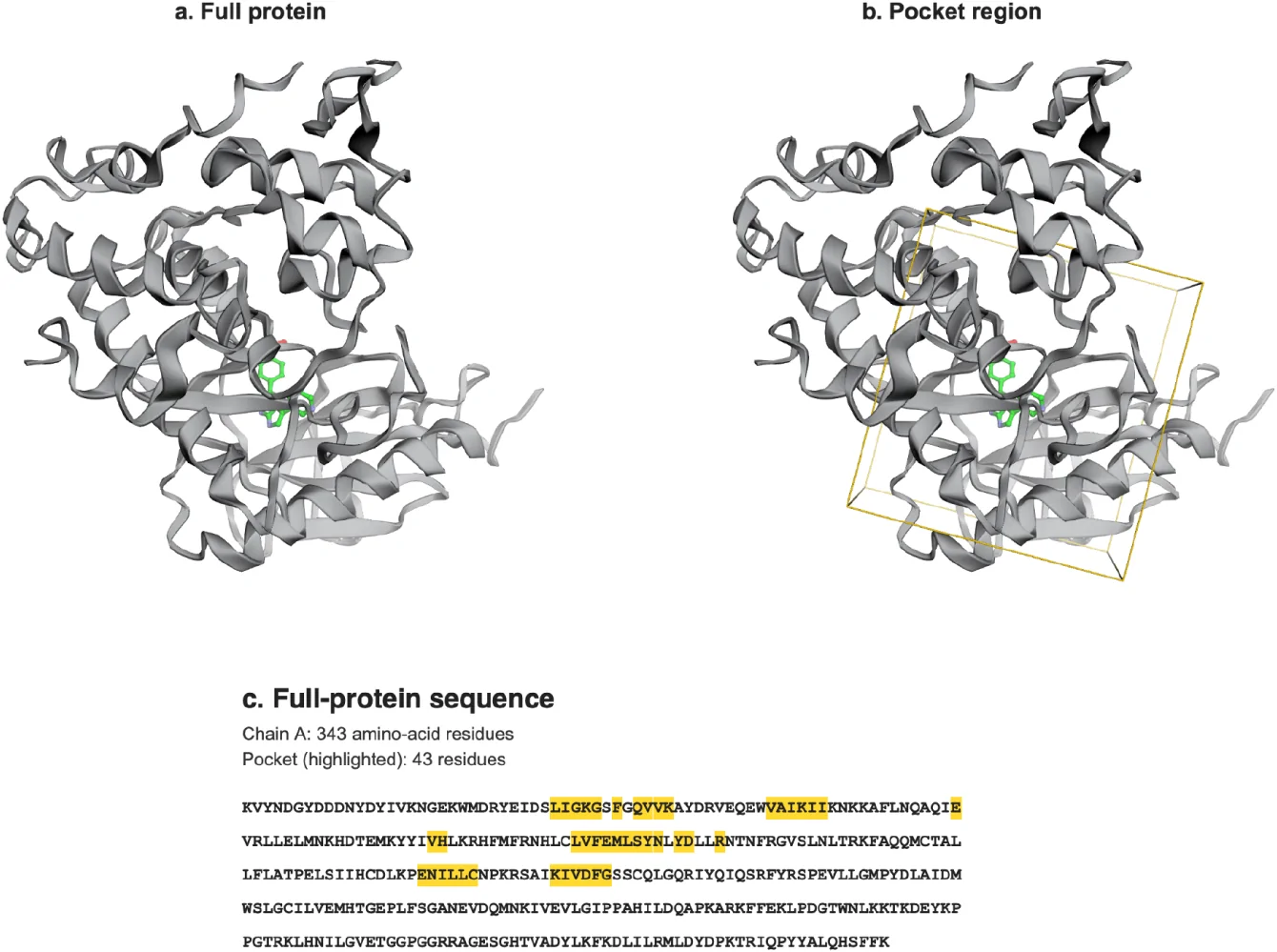
(a) Full protein structure with the bound ligand shown in green. (b) Pocket region enclosed by the yellow box. (c) Full protein sequence, with pocket residues highlighted in yellow.

Using only a short sequence of pocket residues provides limited information about the binding site, capturing residue identity but not full geometry, orientation, electrostatics, or interaction patterns. For this reason, PockLigGPT also explores an external pocket embedding added to the token and positional representations of the decoder. This allows pocket information to guide generation while retaining the simplicity and scalability of a decoder-only architecture.

Thus, PockLigGPT is motivated by three ideas: *molecular generation* can benefit from multistage training; *target conditioning* should focus on the binding-site environment; and *docking-guided RL* can provide an explicit docking-based optimization signal after the model has learned chemical syntax, bioactivity-oriented patterns, and pocket-sequence-conditioned generation. These principles define PockLigGPT as a lightweight pocket-sequence-conditioned molecular language model optimized with docking-guided RL.

## Methods

We adopt the GPT-2 Medium architecture^26^ to build our model. This choice is motivated by the relatively small vocabulary required to encode chemical structures, as it enables an autoregressive transformer with 350M parameters to effectively model chemical syntax and recurring molecular patterns through pretraining. In addition, representing molecules with SELFIES improves the robustness of molecular decoding and reduces the generation of invalid structures,^18^ a property that becomes particularly important during the RL phase, where policy updates can otherwise push the model toward chemically invalid regions of the sequence space.

Figure 2 provides an overview of the PockLigGPT training framework, covering (1) data set filtering and tokenization, (2) sequential pretraining, (3) ligand-only fine-tuning, (4) pocket-sequence-conditioned fine-tuning, and (5) pocket-specific RL optimization.

**Figure 2:**
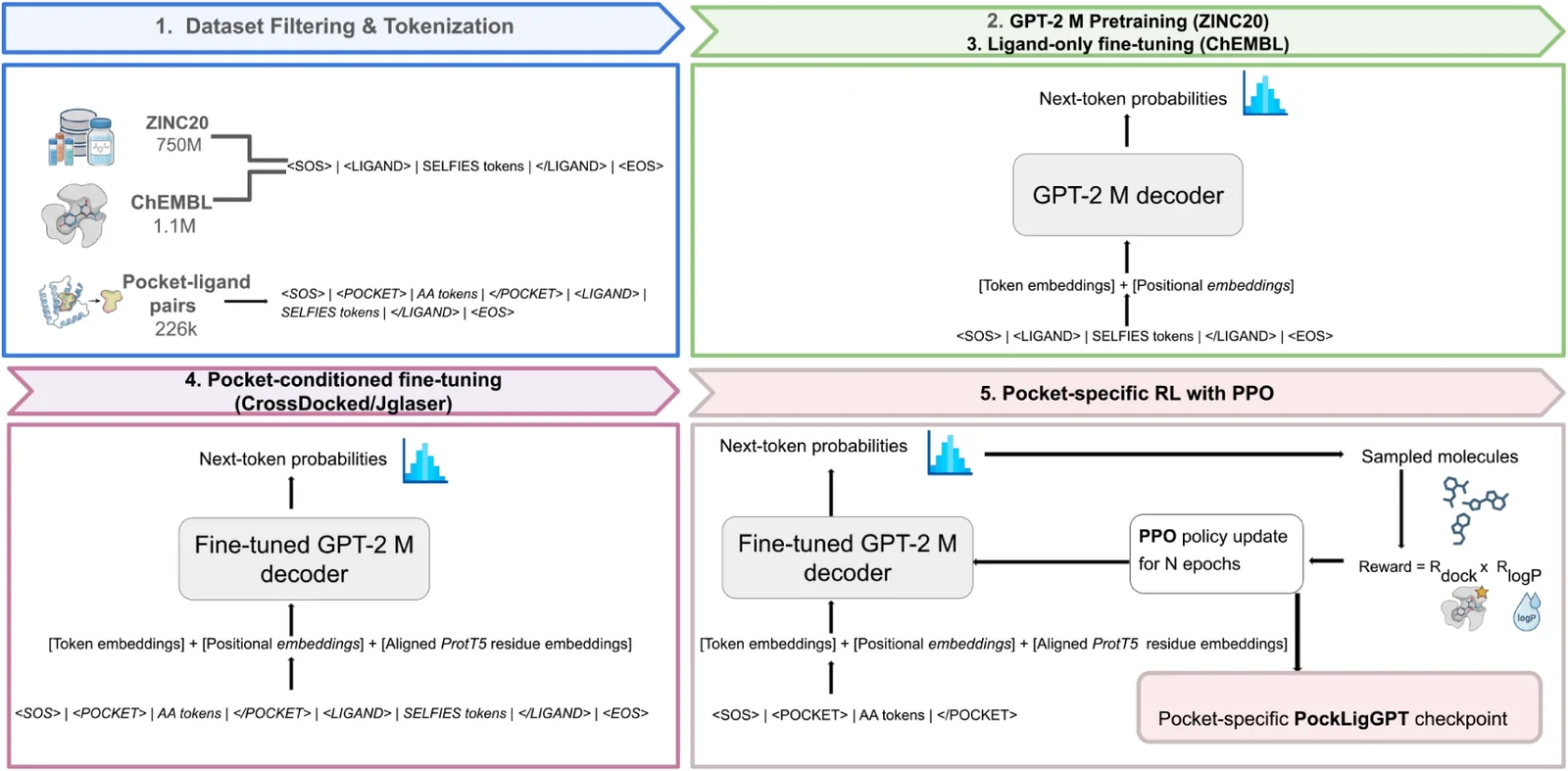
Overview of the PockLigGPT training pipeline. First, ligand-only and pocket–ligand data sets are filtered and tokenized. GPT-2 M is then pretrained on ZINC20 and fine-tuned on ChEMBL. During pocket-conditioned fine-tuning, aligned ProtT5 residue embeddings are added to the token and positional embeddings at pocket-token positions. Finally, pocket-specific reinforcement learning is performed using PPO with a reward combining docking performance and logP regularization, yielding a pocket-specific PockLigGPT checkpoint.

### Data Sets and Filtering Strategy

We used four complementary molecular and pocket–ligand data sources to construct our multistage training pipeline: ZINC20, ChEMBL, CrossDocked, and a Jglaser-derived binding-affinity data set. Each source contributes a distinct type of training information, ranging from large-scale ligand-only chemical pretraining to pocket-sequence-conditioned ligand generation.

#### ZINC20

It was used as the large-scale ligand-only pretraining data set^27^ in phase 2. This database represents the purchasable chemical space, including both in-stock and make-on-demand compounds aggregated from commercial vendor catalogs. Unlike bioactivity databases, ZINC20 is not primarily defined according to experimental target annotations, but through chemical availability and synthetic accessibility. Therefore, it provides a broad chemical prior enriched with realistic and synthetically accessible molecules.

#### ChEMBL

It was used as the ligand-only bioactive fine-tuning data set^28^ in phase 3. In contrast to ZINC20, ChEMBL contains manually curated molecules associated with experimental bioactivity measurements, assays, biological targets, and drug discovery annotations. This stage was intended to shift the broad chemical prior learned from ZINC20 toward the experimentally explored, bioactive, and drug-like chemical space, without yet conditioning generation on specific protein pockets.

#### CrossDocked

It was used as a source of protein pocket–ligand pairs^29^ in phase 4. This data set is derived from experimentally determined protein–ligand complexes from the Protein Data Bank, where ligands are docked into cognate and noncognate receptor structures with similar binding pockets. We used the preprocessed CrossDocked subset in which low-quality poses are removed using an RMSD-based filter and protein pockets are cropped around the ligand-binding region. For each example, we employed the pocket atoms provided in the preprocessed data set. The pocket amino-acid sequence was obtained by selecting the C_α_ atom records in their stored order, mapping the corresponding residue names to one-letter amino-acid tokens, and pairing the resulting sequence with the SELFIES representation of the corresponding ligand.

#### Jglaser-Derived Binding-Affinity Data Set

Additionally, phase 4 used a Jglaser-derived data set containing experimentally characterized protein–ligand pairs with their associated affinity values. This resource integrates information from BindingDB, PDBbind-cn, BioLIP, and BindingMOAD.^30–33^ For each available protein–ligand structure, the pocket was defined by selecting all standard amino-acid residues containing at least one heavy atom within 6.0 Å of any heavy atom of the bound ligand. The selected residues were ordered according to chain identifier and residue number, converted into a one-letter amino-acid sequence, and paired with the SELFIES representation of the corresponding ligand, yielding additional pocket–ligand examples for pocket-sequence-conditioned fine-tuning.

#### Filtering Criteria and Property Control

All the data sets were filtered to maintain a chemically realistic and drug-like training distribution. For ZINC20, molecules were selected from the drug-like region using a calculated logP range of 1–4.5 and a molecular weight range of 250–500 Da.^63^ ChEMBL, CrossDocked, and the Jglaser-derived binding-affinity data set were filtered using Lipinski^64^ physicochemical criteria. Besides, Jglaser pairs were additionally restricted to higher-confidence binders with pChEMBL > 6.5.^65^

This filtering strategy enriches the training distribution with chemically realistic and drug-like molecules. Consequently, the model learns a chemical prior that promotes the generation of compounds with desirable physicochemical properties and synthetic accessibility.

#### Final Filtered Data Sets

The final pretraining set contained approximately 750M ZINC20 molecules. The ChEMBL ligand-only fine-tuning set contained 1.1M Lipinski-compliant molecules. The combined pocket-sequence-conditioned fine-tuning set (CrossDocked + Jglaser) contained 226K pocket–ligand examples. Thus, the training pipeline progressively moves from broad chemical pretraining to bioactivity-oriented adaptation and finally to pocket-context conditioning.

### Tokenizer

All molecules from the filtered data sets were converted to SELFIES and tokenized using a shared vocabulary. The use of a common tokenizer across all training stages ensures that the model can transfer the molecular syntax learned during pretraining to both the ligand-only and the pocket-sequence-conditioned fine-tuning stages. This process resulted in a vocabulary of 469 tokens, which directly determines the dimensionality of the transformer’s output layer. Details about the tokens in our vocabulary are provided in SI–S1.

All ligand-only sequences follow the format
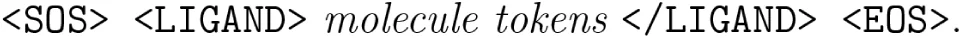


For pocket–ligand pairs, we incorporate pocket-context information by enclosing the pocket-derived amino acid sequence within dedicated pocket tokens. Pocket-sequence-conditioned samples follow the format
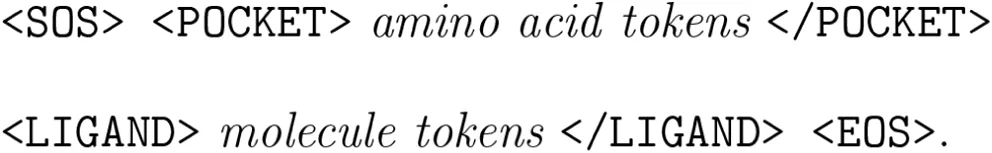


To ensure a uniform sequence length during training, we pad all samples to a block size of 156 tokens using the <PAD> token. This allows us to retain 97.5% of the filtered samples while keeping the computational cost manageable.

### Pretraining on ZINC20

In this phase, we train the GPT-2 Medium model using approximately 750M molecules from the filtered ZINC20 data set. Training follows a standard autoregressive next-token prediction objective over ligand-only SELFIES sequences. It is performed for 10,000 iterations with an effective batch size of approximately 0.5M tokens. At each iteration, molecules are randomly sampled from the data set, so that the model is exposed to approximately 80M distinct molecules throughout training. Although this does not correspond to a full epoch over ZINC20, this stage provides a sufficiently broad exposure to chemical syntax and supports the development of a diverse generative molecular prior.

### Ligand-Only Fine-Tuning on ChEMBL

We fine-tune the pretrained model using 1.1M Lipinski-compliant molecules from ChEMBL. This stage also follows an autoregressive next-token prediction objective over ligand-only SELFIES sequences. Given the size of this data set, we perform full-parameter fine-tuning to adapt the model to the task-specific chemical distribution. This stage encourages the model to capture realistic, bioactive, and drug-like structural patterns commonly associated with experimentally explored chemical matter, refining the model’s underlying chemical distribution before incorporating pocket-context information.

### Pocket-Sequence-Conditioned Fine-Tuning on CrossDocked and Jglaser Binding Affinity Data

To incorporate binding-site information, we represent each pocket as an amino acid sequence enclosed within dedicated pocket tokens and concatenate it with the corresponding ligand SELFIES sequence. We use 226K pocket–ligand pairs to fine-tune the model. The pocket segment is treated as a fixed conditioning context, whereas the ligand segment constitutes the autoregressive prediction target. Consequently, this stage trains the model to predict ligand tokens conditioned on pocket-level information, rather than to predict the pocket sequence itself.

To enrich the pocket representation beyond discrete amino acid tokens, we also use external residue-level embeddings derived from the open-source ProtT5-XL-UniRef50 encoder from the ProtTrans collection.^66^ This is motivated by two observations: first, transformer inputs commonly combine multiple embedding sources, such as token, positional, and segment embeddings;^67^ second, pretrained protein language models provide contextual residue-level representations that encode sequence-derived biophysical, structural, and functional patterns.^66^ Related target-aware molecular generation frameworks also integrate protein language model representations into chemical language models to provide biological context during ligand generation.^61^ We therefore use ProtT5 embeddings as additional fixed conditioning information derived from the pocket sequence, complementing the raw amino acid token sequence. The different strategies used to incorporate pocket information into the transformer architecture are detailed in SI–S5.

At the end of this stage, the model can generate ligand token distributions conditioned on the pocket representation and can therefore be applied to new pocket inputs without additional supervised fine-tuning. However, this sequence-based conditioning alone produces only a limited pocket-specific shift, as discussed in the *Explainability* Section. The model is therefore subsequently refined through pocket-specific RL. Further details of the training pipeline and its computational costs are provided in SI–S2.

### Reinforcement Learning through Proximal Policy Optimization

At this stage, RL is applied independently to each target pocket. During each run, the pocket representation and docking receptor are kept fixed, and the generative policy is optimized toward molecules with improved docking scores while preserving desirable physicochemical properties, following prior work on RL for sequence generation.^68^ Consequently, each RL-optimized checkpoint is specialized to a single pocket rather than forming a single general multipocket RL model. Additional PPO hyperparameters, training-loss curves, reward evolution, and RL computational cost are reported in SI–S4.

#### Reward

Docking scores computed through AutoDock Vina^35^ are widely used as a proxy for binding affinity,^69,70^ but they are based on an empirical scoring function that exhibits known biases. The Vina energy is computed as the weighted sum shown in eq 1

1 $$\begin{array}{ll} E_{\mathrm{Vina}}= & -0.0356\;\mathrm{Gauss}1-0.0052\;\mathrm{Gauss}2+0.8402\;\mathrm{Repulsion}\\ & -0.0351\;\mathrm{Hydrophobic}-0.5874\;\mathrm{Hbonding}+0.0585\;N_{\mathrm{rot}} \end{array}$$

Terms such as the hydrophobic contribution and the penalty on the number of rotatable bonds (*N*_rot_) are part of the Vina scoring function and reflect meaningful aspects of pocket–ligand recognition. However, when Vina is used as the sole optimization signal in a generative RL loop, these terms may introduce exploitable biases. In particular, the hydrophobic contribution can favor molecules with increased global hydrophobicity or larger contact surfaces, while the rotatable-bond term can bias the policy through changes in molecular flexibility. Such changes may improve the docking score without necessarily improving ligand efficiency, selectivity, or drug-like behavior. This motivates the introduction of an additional physicochemical regularization term when using docking as reward. SI–S6 analyzes the individual components of the score and examines the empirical correlations between docking scores and intrinsic physicochemical molecular properties.

#### Docking Component

AutoDock Vina scores typically fall within empirical ranges that depend on the target pocket, commonly between [−12, −6] kcal/mol, although in some cases they are closer to [−10, −4] kcal/mol. Because the empirical score distribution varies across pockets, the rescaling interval must be adapted accordingly. To normalize the reward signal, we apply a linear rescaling to the fixed interval [−6, 6]. Using a fixed raw-score range for all targets can compress the reward signal and produce weak or poorly discriminative rewards, whereas pocket-specific intervals preserve score contrast and support more informative policy updates.

Then, the rescaled score is passed through the logistic function defined in eq 2

2 $$s_{\mathrm{dock}}\left(x\right)=\frac{1}{1+e^{-x}}$$

This transformation maps the normalized docking score to a bounded interval. Because lower docking scores indicate more favorable predicted binding, we invert this signal and apply an exponent γ > 1, as defined in eq 3

3 $$R_{\mathrm{dock}}\left(x\right)={(1-s_{\mathrm{dock}}(x))}^{\gamma }$$

The exponent γ controls the selectivity of the docking reward: values above 1 reduce intermediate and low rewards more strongly than high rewards, thereby increasing the pressure toward molecules with more favorable docking scores. We set γ = 1.3 as a moderate sharpening factor that accentuates high-quality docking predictions without making the reward landscape overly steep. The robustness of this choice was evaluated through a sensitivity analysis reported in SI–S3.

#### LogP Regularization

As mentioned, due to the structure of the Vina scoring function, the RL policy may exploit its hydrophobic and *N*_rot_ terms by generating molecules with excessively high logP values or unnecessary flexibility. To prevent this behavior and preserve drug-like characteristics, we introduce the Gaussian modulation defined in eq 4

4 $$R_{\mathrm{logP}}\left(l\right)=\exp\left(-\frac{{\left(l-\mu \right)}^{2}}{2\sigma^{2}}\right)$$

Here, *l* is the calculated logP, μ = 2.5 is the desired value, and σ = 1.0 controls the penalty strength.

#### Final Reward

Our final RL reward combines both components multiplicatively, as defined in eq 5

5 $$R\left(x,l\right)=R_{\mathrm{dock}}\left(x\right)R_{\mathrm{logP}}\left(l\right)$$

This formulation keeps docking as the primary optimization target, while using logP as a physicochemical gate. The multiplicative form assigns high reward only when both conditions are satisfied: favorable docking and logP within the desired drug-like range. Unlike an additive reward, it limits compensation between terms, reducing the risk that poor docking or excessive hydrophobicity is masked by the other component. Sensitivity analyses comparing additive and multiplicative formulations, as well as different logP modulation parameters, are provided in SI–S3.

### Computational Validation: Docking Studies

#### Ligand Preparation

Three virtual chemical libraries comprising 256 potential hit inhibitors per target pocket were used in SMILES format. These SMILES representations were converted into Maestro format using the *structconvert* utility from the Schrödinger suite.^71^ Ligand preparation was performed using the LigPrep module within the Maestro platform.^72–75^ LigPrep expands tautomeric and ionization states, ring conformations, and stereoisomers to generate relevant three-dimensional representations of each molecule for subsequent Glide docking.

#### Protein Preparation

The protein structures used for computational analysis were obtained from the Protein Data Bank with PDB IDs 6EIF,^76^ 7Q1P,^77^ and 5TOL,^78^ corresponding to DYRK1A, BuChE, and BACE-1, respectively. Protein preparation was performed using the Protein Preparation Workflow^79,80^ available within the Maestro suite.^73^ The workflow included initial preprocessing steps such as bond order assignment and structural refinement, assisted by Prime.^81–83^ Hydrogen bond optimization was carried out at pH 7.2 using PROPKA,^84^ based on p*K*_a_ predictions and subsequent evaluation of hydrogen-bonding patterns and associated desolvation penalty scores. The preparation protocol concluded with a restrained energy minimization using the OPLS4 force field.^85^

#### Docking Studies Using Glide

Docking simulations were performed with the Glide extra precision (XP) mode available within the Schrödinger software suite,^86–89^ without imposing docking constraints. This choice was made to evaluate whether molecules optimized with AutoDock Vina also received favorable docking scores under an independent docking engine, thereby reducing the risk of reporting candidates that only exploit the Vina scoring function. This follows the rationale of consensus and cross-docking strategies, where agreement across independent docking programs can increase confidence in predicted binding modes and reduce single-engine scoring bias.^90–92^

The docking grid was established by centering it on the geometric center of the crystallographic ligand for each target. The reference ligands were B5T, 8UW, and 7H3 for DYRK1A, BuChE, and BACE-1, respectively. For grid preparation, a van der Waals radius scaling factor of 1.0 and a partial charge cutoff of 0.25 were used. Default settings were applied for ligand handling, including flexible sampling and the incorporation of Epik state penalties in the docking score. Finally, postdocking minimization was performed using default parameters.

Poses and docking scores are shown in Figure 3 and Table S1 in the Supplementary Data.

**Figure 3:**
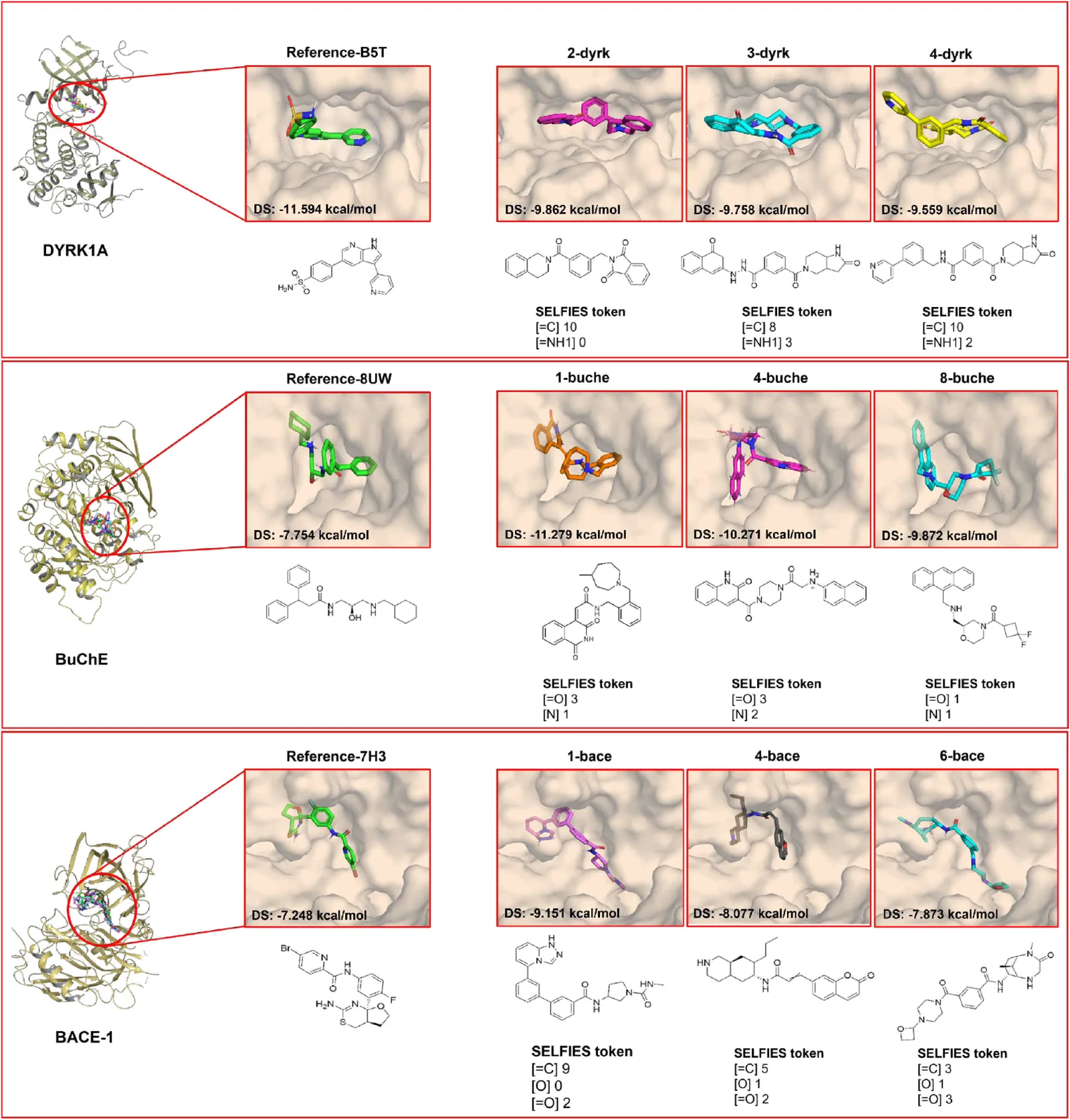
Docking poses of three representative top-ranked molecules and corresponding crystallographic reference ligands for DYRK1A (top), BuChE (middle), and BACE-1 (bottom). Docking scores and selected SELFIES token counts enriched through RL are also shown and further analyzed in Section *Explainability*.

## Results

We evaluated PockLigGPT through four analyses: ablation of the multistage training pipeline, benchmarking against pocket-sequence-conditioned baselines, docking assessment on Alzheimer’s disease-associated targets, and token-level analysis of RL-induced changes in the generative policy. All quantitative analyses were assessed using the following metrics:

- *Docking score (kcal/mol).* AutoDock Vina docking score, where lower values indicate more favorable predicted binding poses. MGLTools was used for structure preparation prior to Vina docking.
- *QED.* Quantitative estimate of drug-likeness, ranging from 0 to 1.^63^
- *LogP.* Octanol–water partition coefficient, used as a measure of molecular lipophilicity. RDKit MolLogP^93^ was used to estimate logP values, with the desired drug-like interval set to 0–5.
- *SAS.* Synthetic Accessibility Score, ranging from 1 (easy to synthesize), to 10, (difficult).^94^
- *Molecular diversity.* Defined as 1 – *S̅*, where *S̅* is the mean pairwise Tanimoto similarity over ECFP4 fingerprints.^95^
- *Validity, uniqueness, and novelty.* Standard generation metrics used to evaluate whether the produced molecules are chemically valid, nonduplicated, and absent from the reference training set.

### Ablation of Pocket-Sequence-Conditioned Fine-Tuning and Docking-Guided RL

To assess the contribution of each of the 4 stages in the proposed training pipeline, we compared five model variants: the ZINC20-pretrained baseline; the ligand-only model fine-tuned on ChEMBL; the model subsequently fine-tuned on pocket–ligand pairs; the pocket-specific models obtained by independently applying docking-guided RL to each evaluation pocket; and, as a control, the ligand-only ChEMBL-fine-tuned model further optimized with the same pocket-specific RL procedure but without pocket-sequence conditioning. All checkpoints were evaluated on three Alzheimer’s disease-associated binding pockets: DYRK1A (PDB: 6EIF),^36^ BuChE (PDB: 7Q1P),^37^ and BACE-1 (PDB: 5TOL).^38^ This analysis allows us to disentangle the individual contributions of general chemical pretraining, bioactivity-oriented ligand-only adaptation, pocket-sequence-conditioned fine-tuning, and explicit pocket-specific docking-guided optimization in a disease-relevant setting.

Table 1 shows a progressive improvement in docking performance across the proposed multistage pipeline. The ZINC-pretrained baseline already generates valid, unique, and novel molecules with favorable drug-like profiles, reflecting the value of large-scale chemical pretraining. However, its median Vina score remains limited compared with later stages. Ligand-only fine-tuning on ChEMBL slightly improves the median docking score, suggesting that exposure to bioactive chemical space helps move the model toward molecules with stronger predicted docking interactions. Nevertheless, this stage also reduces QED and increases the median logP, indicating that bioactivity-oriented data must be carefully curated to avoid degrading physicochemical quality.

**Table 1:** Ablation of the Multistage Training Strategy across Docking and Molecular Quality Metrics^a^

**Method**	**Vina Score** (kcal/mol, ↓)	**QED (↑)**	**logP** (target 0–5)	**SAS (↓)**	**Diversity (↑)**	**Validity** (%, ↑)	**Uniqueness** (%, ↑)	**Novelty** (%, ↑)
ZINC20 pretraining	–7.90 ± 0.50	0.76 ± 0.01	2.44 ± 0.08	3.12 ± 0.67	0.87	100.00 ± 0.00	100.00 ± 0.00	100.00
Ligand-only FT	–8.08 ± 0.40	0.63 ± 0.01	3.27 ± 0.12	3.11 ± 1.05	0.89	100.00 ± 0.00	100.00 ± 0.00	99.48
Pocket–ligand FT	–8.45 ± 0.35	0.52 ± 0.02	3.72 ± 0.04	3.70 ± 1.12	0.89	100.00 ± 0.00	100.00 ± 0.00	99.61
Ligand-only FT + RL	–9.26 ± 0.25	0.74 ± 0.06	2.55 ± 0.13	2.27 ± 0.16	0.80	100.00 ± 0.00	89.19 ± 1.30	99.60
Pocket–ligand FT + RL	–9.90 ± 0.30	0.73 ± 0.03	2.95 ± 0.23	3.53 ± 1.03	0.84	100.00 ± 0.00	87.76 ± 3.63	100.00

a For each method, *n* = 256 molecules were generated and evaluated per receptor pocket. Reported values correspond to the mean ± standard deviation of the pocket-level medians across the three receptor pockets. The ligand-only RL control was optimized independently for each receptor without pocket conditioning. ↑ Higher is better. ↓ Lower is better.

Pocket–ligand fine-tuning further improves the median Vina score, from −8.08 to −8.45 kcal/mol. This suggests that introducing pocket–ligand information guides the model toward generating structures that are more compatible with binding-site environments. However, this improvement is accompanied by a decrease in QED and an increase in SAS, showing that supervised pocket-sequence-conditioned fine-tuning alone is not sufficient to guarantee sufficiently good molecular quality. This result supports the need for an additional optimization stage.

The largest improvement is obtained after docking-guided RL. The final pocket-conditioned model achieves the best median docking score, improving from −8.45 to −9.90 kcal/mol, while recovering a high QED value and maintaining a high fraction of molecules within the desired logP range. Importantly, the ligand-only model optimized using the same pocket-specific RL procedure, but without pocket-sequence conditioning, reaches a median Vina score of only −9.26 kcal/mol. This comparison indicates that, although docking-guided RL substantially improves both models, pocket-sequence conditioning provides an additional pocket-specific advantage beyond that achieved through direct docking optimization alone.

Note that although QED and SAS are not explicitly included in the reward, their improvement can be explained through indirect associations between the optimized objectives and molecular physicochemical properties. In particular, Figure S11 in SI–S6 shows that logP is positively correlated with molecular weight (*r* = 0.35), whereas molecular weight is negatively correlated with QED (*r* = −0.37). Therefore, constraining logP toward a moderate range may indirectly limit the generation of excessively large molecules and help preserve higher QED values.

The improvement in SAS seems to arise through a different association. Docking score is positively correlated with SAS (*r* = 0.30); because lower, more negative docking scores indicate more favorable predicted binding, this means that improved docking tends to coincide with lower SAS values. These changes should therefore be interpreted as emergent effects of optimizing coupled molecular properties, together with the learned chemical prior, rather than as the direct optimization of QED or SAS. This indicates that RL is the main driver of docking-oriented optimization. Importantly, the improvement in docking does not lead to a collapse in validity or novelty, both of which remain at 100%. However, uniqueness decreases to 87.76%, suggesting that RL concentrates the model distribution around high-reward regions of chemical space. This behavior is expected in goal-directed molecular optimization and highlights the importance of balancing exploitation with diversity-preserving exploration mechanisms.

Together, these results show that each of the proposed stages truly contributes to progressively improving binding-oriented generation, with docking-guided RL providing the strongest gain while maintaining acceptable molecular quality.

### Benchmarking against Pocket-Conditioned Baselines

We benchmarked PockLigGPT against crystallographic reference ligands and three representative pocket-conditioned baselines: AR,^8^ Pocket2Mol,^7^ and TargetDiff,^9^ following the evaluation protocol adopted in TargetDiff. AR is an autoregressive graph neural network-based model that generates 3D ligands by sequentially placing atoms inside the protein pocket. Pocket2Mol is an E(3)-equivariant autoregressive model that preserves geometric consistency under rotations, translations, and reflections while jointly predicting frontier atoms, atomic positions, atom types, and chemical bonds during pocket-conditioned generation. TargetDiff is an equivariant diffusion model that jointly generates ligand atom coordinates and atom types conditioned on the 3D protein pocket. Baseline molecules and reference ligands were obtained from the TargetDiff evaluation files and reevaluated using the same docking, filtering, and molecular-property pipeline applied in PockLigGPT. These methods were selected because their outputs were available and could be assessed under a common and reproducible protocol.

We also considered additional pocket-based generative models. However, several could not be included in the main comparison because their code was unavailable, incomplete, or could not be reliably reproduced in our computational environment. Lingo3DMol was evaluated on the subset of pockets for which molecule generation was successful, and the corresponding results are reported in SI–S7. Because outputs were not available for all 10 pockets, Lingo3DMol was not included in the main quantitative table.

Since molecular generation followed by docking-based evaluation is computationally expensive, as described in SI–S2, all methods were evaluated on a random subset of 10 pockets selected from the TargetDiff test set, not included in the supervised pocket–ligand fine-tuning set. When RL is subsequently applied to a fixed pocket, the resulting model is specialized to that target and is no longer considered zero-shot for that pocket.

For these targets, the pocket was constructed from a known bound ligand, by selecting residues containing at least one heavy atom within 6.0 Å of any ligand heavy atom. The selected residues are ordered by chain identifier and residue number, converted into a one-letter amino-acid sequence, and processed using the same ProtT5 conditioning pipeline.

For each method, molecules were assessed employing the same docking, filtering, and molecular-property evaluation protocol. However, the methods were not necessarily optimized using the same objective: PockLigGPT includes a pocket-specific RL stage based on an AutoDock Vina-derived reward, whereas the baseline methods were generated according to their original training objectives and not directly optimized against Vina.

Table 2 shows that PockLigGPT achieved the best median docking score among the evaluated methods on the selected receptor subset. It also showed the lowest standard deviation in docking score, suggesting more consistent behavior across pockets. Importantly, this docking-oriented improvement was accompanied by favorable molecular quality. PockLigGPT achieved the highest QED, the lowest SAS, and median logP values within the desired range, with reasonably good performance in the remaining indicators.

**Table 2:** Model Evaluation across Docking and Molecular Quality Metrics on the Selected Receptor Set^a^

**Method**	**Vina Score** (kcal/mol, ↓)	**QED (↑)**	**logP** (target 0–5)	**SAS (↓)**	**Diversity (↑)**	**Validity** (%, ↑)	**Uniqueness** (%, ↑)	**Novelty** (%, ↑)
Reference	–7.96 ± 2.64	0.43 ± 0.21	–0.39 ± 4.09	3.39 ± 1.03	–	100.00 ± 0.00	100.00 ± 0.00	–
AR	–7.08 ± 2.07	0.42 ± 0.10	0.03 ± 1.64	4.63 ± 0.94	0.83 ± 0.08	100.00 ± 0.00	100.00 ± 0.00	100.00 ± 0.00
Pocket2Mol	–8.21 ± 3.25	0.55 ± 0.12	1.62 ± 1.50	3.27 ± 0.88	0.86 ± 0.02	100.00 ± 0.00	100.00 ± 0.00	100.00 ± 0.00
TargetDiff	–8.19 ± 2.22	0.38 ± 0.13	1.08 ± 1.95	4.74 ± 0.80	0.87 ± 0.01	100.00 ± 0.00	98.07 ± 4.62	100.00 ± 0.00
PockLigGPT	–8.38 ± 0.98	0.75 ± 0.03	2.89 ± 0.35	2.92 ± 0.37	0.84 ± 0.02	100.00 ± 0.00	95.07 ± 3.49	100.00 ± 0.00

a For each method, *n* = 100 molecules were evaluated per receptor pocket. Reported values correspond to the mean ± standard deviation of the pocket-level medians across the 10 receptor pockets. ↑ Higher is better. ↓ Lower is better.

Overall, this benchmark suggests that PockLigGPT provides a favorable trade-off between docking performance and chemical plausibility on the selected receptor subset. Although it does not directly generate 3D coordinates inside the pocket, it remains competitive with respect to 3D pocket-conditioned baselines under the TargetDiff evaluation protocol, while producing molecules with stronger drug-like profiles. A pocket-level analysis of this benchmark is provided in SI–S7.

### Docking Studies

The top ten molecules generated for each target protein (DYRK1A, BuChE, and BACE-1) were subsequently evaluated through docking studies to analyze their interactions with catalytic and key residues. In addition, the same docking protocol was applied to the corresponding reference compounds for comparison (see Section *Methods*). The best generated molecules and their docking poses are shown in Figure 3, while the corresponding docking scores are reported in Table S1 in the Supplementary Data.

- *DYRK1A Inhibitors*. We found that the reference compound forms hydrogen bonds with Glu239 and Leu241 and has an arene group oriented toward Lys188, as described by Czarna et al.^76^ Among the top ten compounds, three of them (3-dyrk, 4-dyrk, and 9-dyrk) established hinge-binding hydrogen bonds with Glu239 (gatekeeper +1) and Leu241 (gatekeeper +3),^96^ while also interacting with the catalytic residue Lys188. These compounds achieved docking scores ranging from −9.76 to −8.86 kcal/mol, comparable to the score of the reference compound. Additionally, three candidates (2-dyrk, 8-dyrk, and 10-dyrk) formed at least two hydrogen bonds with these key residues. Finally, two compounds (5-dyrk and 7-dyrk), whose arene rings were oriented toward Lys188, also formed at least one hydrogen bond with either Glu239 or Leu241.
- *BuChE Inhibitors*. The reference compound interacts with the catalytic site formed by Ser198 and His438. Interestingly, the top ten molecules were positioned above the catalytic site, consistent with the positioning reported by the authors who originally published the structure, potentially occluding access to substrate molecules.^77^ Seven of the ten compounds formed interactions with the catalytic residue His438, yielding docking scores ranging from −9.66 to −11.28 kcal/mol, notably more favorable than that of the reference compound (−7.75 kcal/mol).
- *BACE-1 Inhibitors*. The crystallized compound (7H3) contains a biaryl amide moiety, as found in BACE-1 inhibitors such as verubecestat,^97^ and forms interactions with the catalytic aspartates. Besides, the amide NH forms a hydrogen bond with the backbone carbonyl oxygen of Gly230.^78^ Notably, the compound ranked first for BACE-1 features a biaryl amide–like scaffold, with a docking score of −9.15 kcal/mol compared to −7.25 kcal/mol for the reference compound. Thirty percent of the top ten compounds showed interactions with the catalytic aspartates Asp32 and Asp228. The docking scores obtained for these compounds were more favorable than that of the reference compound. In addition, five compounds formed favorable hydrogen bonds with Gly230 (two of them also interacting with the catalytic aspartates), a residue located near Asp228 that contributes to the S1 and S3 subpockets.

### Explainability

We next investigate how target-dependent chemical patterns emerge during training by comparing the effects of pocket conditioning alone and pocket conditioning followed by RL. Molecular generation is conditioned on a textual pocket representation, while structural constraints are introduced indirectly through the docking-based reward used during RL. Consequently, the observed chemical specialization reflects a combination of pocket-dependent priors and reward-driven optimization.

To quantify changes in chemical composition, we analyze shifts in SELFIES token frequencies across training stages. The statistical significance of token frequency differences is assessed using per-token proportion tests with Benjamini–Hochberg^98^ false discovery rate correction (FDR < 0.05). Only tokens passing this threshold are shown in the corresponding figures. Importantly, our explainability analysis should be understood as a policy-level characterization of how the generative distribution evolves during optimization. The structural relevance of these shifts is further supported by the docking pose analysis described in Section *Docking Studies*, which provides an orthogonal structure-based perspective on the binding hypotheses for the optimized candidates.

#### Effect of Pocket-Sequence Conditioning

The analysis of pocket amino acid composition reveals clear differences between the three targets. DYRK1A contains a relatively higher proportion of charged and polar residues, compatible with a compact binding environment where directional interactions may be favored. In contrast, BuChE and BACE-1 display increased enrichment in aromatic residues and glycine-rich segments, suggesting broader and more conformationally permissive binding environments.

Consistent with these differences, conditioning on pocket sequences induces measurable shifts in the generated ligand token distributions. As Figure 4 shows, DYRK1A-conditioned generations show enrichment in unsaturated SELFIES tokens such as [=C] and [=N], as well as nitrogen-containing functionalities such as [NH1], suggesting an increased tendency to sample conjugated heteroaromatic fragments. In contrast, BuChE- and BACE-1-conditioned generations are enriched in aliphatic carbon tokens including [C@H1] and [C@@H1], typically associated with increased conformational flexibility. Notably, BACE-1 conditioning also promotes enrichment of branching motifs ([Branch1]), consistent with the generation of more topologically complex and adaptable scaffolds.

**Figure 4:**
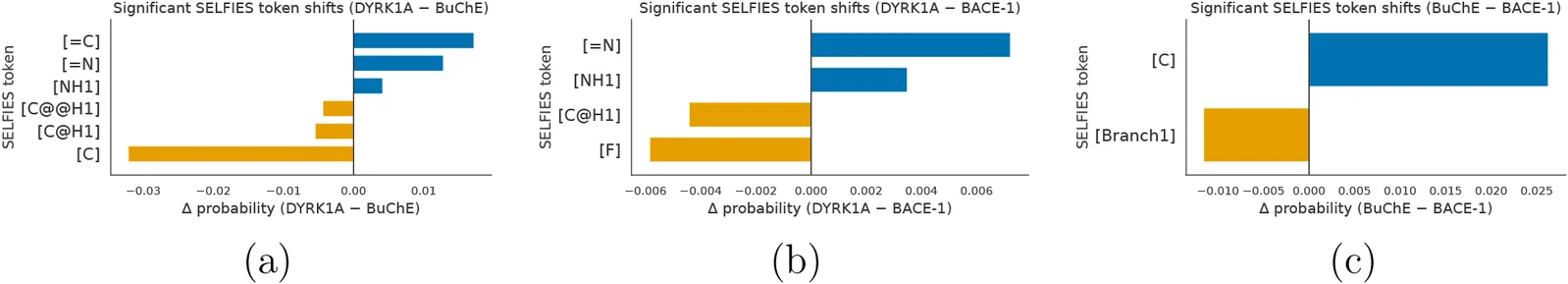
SELFIES token shifts induced by pocket conditioning across three protein-pair comparisons: (a) DYRK1A *vs* BuChE, (b) DYRK1A *vs* BACE-1, and (c) BuChE *vs* BACE-1.

Overall, pocket conditioning seems to provide a contextual prior that guides the initial generative distribution according to broad pocket properties, such as polarity and flexibility, but does not by itself enforce strong pocket-specific binding strategies. More pronounced specialization actually emerges after RL, when docking feedback introduces an explicit optimization objective.

#### Effect of Pocket-Sequence Conditioning Combined with RL

After RL, the generative policy enters a distinct regime with reward-based feedback driving systematic and statistically significant changes in token distributions. These shifts reflect both the structure of the AutoDock Vina scoring function and the explicit lipophilicity constraint included in the reward.

Across all three targets, Figure 5 shows consistent enrichment of tokens associated with increased scaffold rigidity and reduced conformational freedom, most prominently [=C] and [Ring1]. This indicates that RL generally favors more preorganized molecular architectures, reducing entropic penalties and improving geometric complementarity under docking evaluation. In parallel, heteroatom-containing tokens including [=N], [=O], and [NH1] are enriched across multiple targets, supporting the introduction of additional hydrogen-bonding capacity and polar anchoring sites.

**Figure 5:**
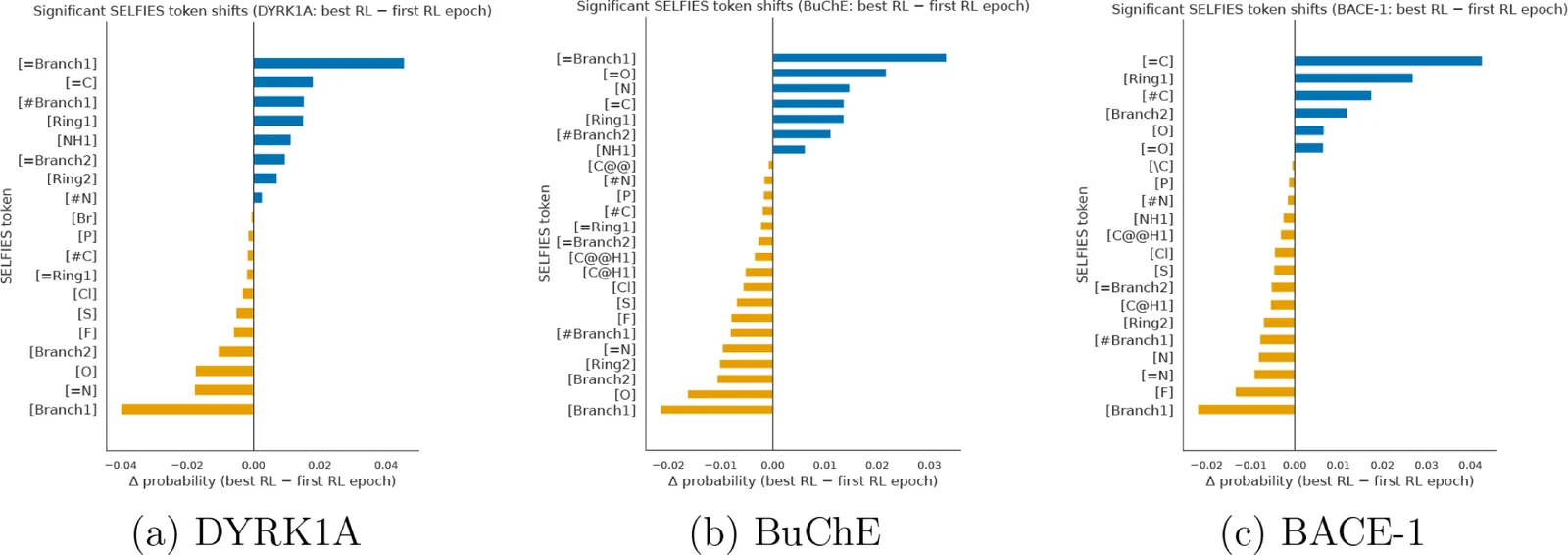
SELFIES token shifts induced by RL. The plots show differences between first and best epoch for (a) DYRK1A, (b) BuChE, and (c) BACE-1.

Conversely, branching tokens such as [Branch1], as well as halogen and heavier heteroatom tokens (e.g., [F], [Cl], [S], and [P]) are systematically depleted after RL. This depletion likely reflects a combined effect of limiting conformational energetic costs under docking and satisfying the lipophilicity constraint imposed by the reward formulation. These trends are consistent with the docking results in Section *Docking Studies*, where optimized ligands typically display higher conjugation and more compact scaffolds, often accompanied by increased polar functionality.

Although the three targets exhibit distinct pocket characteristics, RL induces partially convergent chemical trends, suggesting that docking-driven optimization promotes a transferable set of structural heuristics (rigidity, heteroatom density, reduced branching) that are broadly beneficial under the scoring function, while still allowing for target-dependent variations.

#### Structural Evolution of Representative Ligands during RL

To connect token-level shifts with concrete molecular structures, we examine representative ligands generated for each target before and after RL optimization (Figure 6). Unlike the top-ranked molecules in Figure 3, these examples are selected to illustrate structural evolution during RL rather than to report the best-scoring candidates. These examples provide a qualitative interpretation of how the statistical enrichment patterns translate into changes in representative molecular structures. Importantly, these structural trends can be interpreted in light of the docking-derived interaction hypotheses described in Section *Docking Studies*, providing complementary evidence that the enriched chemical motifs are compatible with plausible binding modes for each target.

**Figure 6:**
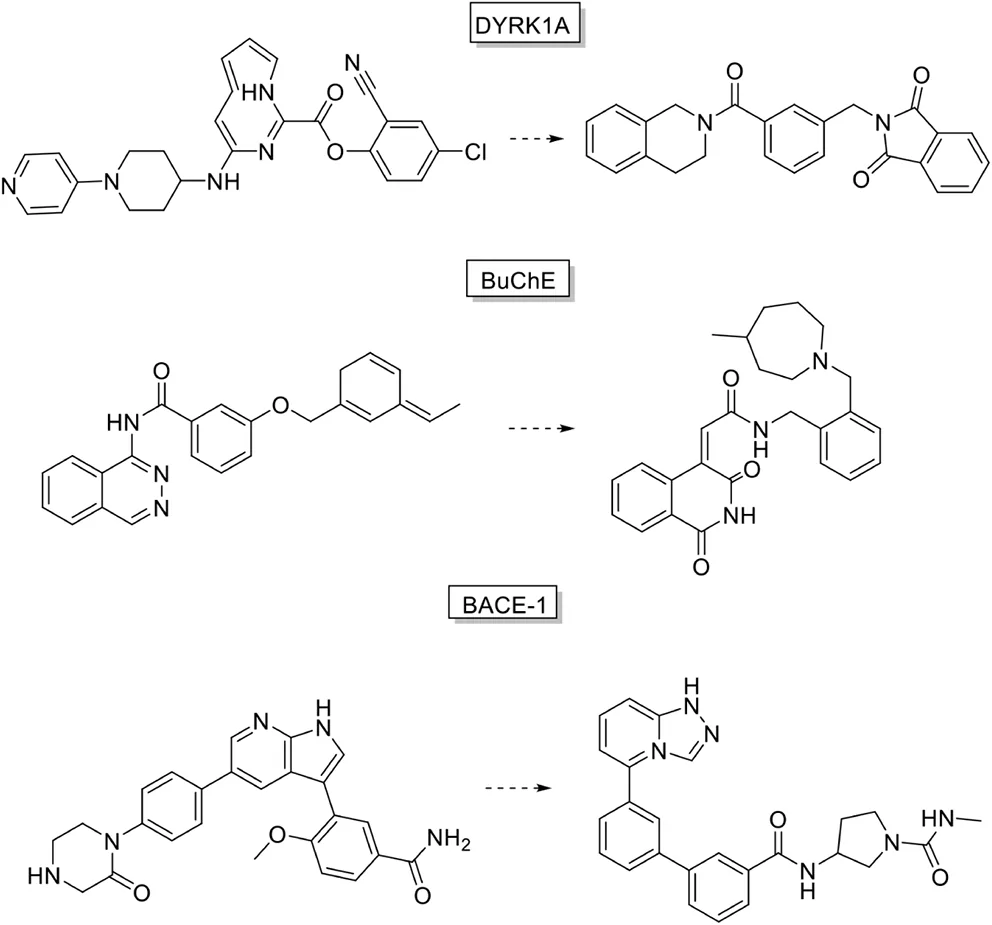
Representative ligands generated before and after RL optimization. Rows correspond to DYRK1A, BuChE, and BACE-1, respectively. Arrows indicate comparison between pre-RL and post-RL representative molecules.

The top row of Figure 6 illustrates a structural transition in DYRK1A-directed ligands before and after RL optimization. The pre-RL compound contains an aromatic/heteroaromatic framework but incorporates a relatively flexible side chain featuring a piperazine moiety and multiple rotatable single bonds, which increases conformational freedom. After RL, the optimized ligand adopts a more scaffold-centered architecture characterized by a higher density of fused and polycyclic ring systems. Although the post-RL ligand still contains rotatable linker segments (e.g., methylene–amide connections), the overall structure appears more rigid and ring-dense, consistent with the enrichment of rigidity-associated SELFIES tokens observed after RL.

This evolution suggests that RL promotes more preorganized kinase-like architectures that may improve docking performance through reduced conformational freedom and increased shape complementarity. This interpretation is consistent with the DYRK1A docking poses discussed in Section *Docking Studies*, where optimized candidates exhibit interaction patterns compatible with hinge-region binding determinants.

For BuChE, the middle row of Figure 6 shows that the pre-RL ligand already exhibits an aromatic scaffold with polar anchoring functionalities, including an amide and ether linkage, which is compatible with the large and permissive BuChE gorge. After RL, the optimized ligand shifts toward a more ring-dense architecture, featuring a rigid imide-containing core combined with a bulky saturated cyclic amine substituent. This indicates that RL does not necessarily enforce maximal conjugation, but rather promotes structural motifs that combine rigid anchoring elements with flexible saturated components.

Such architectural balance is consistent with BuChE’s ability to accommodate multiple binding modes and allows docking improvements through cumulative hydrophobic packing and polar contacts rather than a single dominant interaction. This agrees with the docking analysis in Section *Docking Studies*, where high-scoring BuChE candidates frequently occupy the gorge region in poses that plausibly occlude substrate access.

For BACE-1, the bottom row of Figure 6 shows that the pre-RL ligand already contains aromatic and heteroaromatic systems, as well as polar functional groups including an amide and a cyclic amine functionality, indicating that ionizable and hydrogen-bonding features are present even before optimization. After RL, the optimized ligand retains a comparable aromatic framework but introduces a more structured polar motif characterized by additional amide/urea-like carbonyl functionality and a substituted cyclic amine fragment.

Rather than reflecting a simple gain of ionizable nitrogen, the post-RL structure suggests a refinement toward functional group arrangements that can support more directional hydrogen bonding and polar anchoring, consistent with the requirements of the highly polar BACE-1 catalytic pocket. This interpretation aligns with the docking poses described in Section *Docking Studies*, where optimized BACE-1 candidates exhibit binding modes compatible with the polar environment of the active site.

## Conclusions

This work has introduced PockLigGPT, a pocket-sequence-conditioned molecular language model for *de novo* ligand generation. The model combines large-scale chemical pretraining on ZINC20, bioactivity-oriented adaptation on ChEMBL, pocket-sequence-conditioned fine-tuning on pocket–ligand pairs, and docking-guided RL. This staged strategy progressively guides the model from general chemical syntax toward bioactive, pocket-aware, and docking-oriented molecular generation.

Our results show that intermediate fine-tuning stages are important before applying docking-based optimization. ChEMBL adaptation and pocket–ligand fine-tuning help shape the chemical distribution before RL, while the final docking-guided RL provides the strongest improvement in docking performance. However, because docking scores are empirical and can be exploited by generative models, physicochemical regularization seems necessary to preserve drug-like molecular profiles.

Across the evaluated benchmark, PockLigGPT achieved competitive docking performance against pocket-conditioned baselines while maintaining high QED, low synthetic accessibility scores, and a large fraction of molecules within the desired logP range. These results suggest that a lightweight sequence-based model can remain competitive with 3D pocket-conditioned generators without directly generating fixed 3D coordinates.

We also found that conditioning on pocket amino acid sequences alone induces moderate target-dependent shifts, whereas docking-guided RL produces stronger and more interpretable changes in the generative policy. Token-level analyses showed enrichment and depletion of specific SELFIES tokens after RL, indicating that the model learns chemically meaningful adaptations in response to the docking-based reward.

A remaining challenge is target specificity. Docking-based rewards may improve scores across multiple targets, which can be useful for multitarget design but problematic when selectivity is required. Future work should therefore explore reward functions that jointly optimize docking performance, selectivity, physicochemical quality, and synthetic feasibility.

Overall, PockLigGPT demonstrates that molecular language modeling, pocket-sequence conditioning, and docking-guided RL can be integrated into a practical pipeline for *de novo* drug design.

## Supplementary Material





## Data Availability

The data sets used to train the models are publicly available. ZINC20 is accessible at https://zinc.docking.org/, the CrossDocked data set at https://drive.google.com/drive/folders/1j21cc7-97TedKh_El5E34yI8o5ckI7eK, and the Jglaser Binding Affinity data set at https://huggingface.co/datasets/jglaser/binding_affinity. Code and model weights are publicly available at https://github.com/pvaras8/PockLigGPT_official.git.

## Notes

S1. Tokenizer analysis; S2. Model hyperparameters, training losses, and computational cost; S3. Reward analysis; S4. RL analysis; S5. Types of conditioning strategies; S6. Docking formula analysis; S7. Pocket-level benchmark analysis; S8. Top-ranked molecules and docking analysis; S9. Mathematical formulation of pocket-sequence conditioning (PDF)
Supplementary Data Table S5. Pocket–ligand interaction profiles for the top-ranked generated molecules and the crystallographic reference ligands in DYRK1A, BuChE, and BACE-1 binding pockets (XLSX)
