# Supplementary material for: PockLigGPT: Pocket-Sequence-Conditioned Molecular Generation with GPTs and RL
Source: J Chem Inf Model. 2026 Aug 17;66(17):10847–61. doi: 10.1021/acs.jcim.6c01980 (PMC13580112; doi:10.1021/acs.jcim.6c01980)
Supplement: Supplementary file 1 [file ci6c01980_si_001.pdf]

Supplementary Information

PockLigGPT: Pocket-Sequence-Conditioned Molecular Generation with GPTs and RL

Pablo Varas Pardo[1,2,3], Guillermo Marcos-Ayuso[1], Eugenia Ulzurrun[4], David Ríos Insua[2], Nuria E. Campillo[4,*]

[1] AItenea Biotech S.L., Madrid 28014, Spain
[2] Instituto de Ciencias Matemáticas (ICMAT-CSIC), 28049 Madrid , Spain
[3] Universidad Autónoma de Madrid, Escuela de Doctorado, 28049 Madrid, Spain
[4] Centro de Investigaciones Biológicas Margarita Salas (CIB Margarita Salas-CSIC), 28040 Madrid, Spain

[*] Corresponding author:
nuria.campillo@csic.es

S1 Tokenizer analysis

Table S1 summarizes the composition of the tokenizer vocabulary. During subsequent training stages, certain tokens become more frequent than others, and the proposed model learns to exploit token patterns that correlate with higher rewards.

Table S1: Composition of tokenizer vocabulary.

**Category**	**#tokens**	**Example tokens**	**Purpose**
Special control tokens	4	`<SOS>`, `<EOS>`, `<PAD>`, `<UNK>`	Sequence control and padding
Ligand delimiters	2	`<LIGAND>`, `</LIGAND>`	Explicit ligand boundary markers
Pocket delimiters	2	`<POCKET>`, `</POCKET>`	Protein pocket boundary markers
Amino acid tokens	20	`<AA_ALA>`, `<AA_GLY>`, `<AA_TYR>`	Residue-level pocket sequence encoding
Branching operators	8	`[Branch1]`, `[#Branch2]`	Open molecular branches
Ring closures	13	`[Ring1]`, `[-/Ring2]`	Close molecular rings
Atomic and bond tokens	101	`[C]`, `[=O]`, `[/N]`	Atom identity and bond order
Stereochemistry	61	`[C@]`, `[C@@H1]`	Chiral configuration
Chemical state modifiers	258	`[N+1]`, `[CH1]`, `[13C]`, `[18F]`	Charge, hydrogens, isotopes, and oxidation states

S2 Model hyperparameters, training losses, and computational cost

Model hyperparameters. Table S2 summarizes the architectural and training hyperparameters of the proposed model.

Table S2: Architecture and training hyperparameters.

Hyperparameter	Value
Model variant	GPT-2 M
Number of layers (n_{layer})	24
Hidden dimension (d_{model})	1024
Attention heads	16
Sequence length	156
Dropout	0.1
Batch size per GPU	32
Number of GPUs	4
Gradient accumulation steps	104
Effective batch size (tokens)	524 288
Optimizer	AdamW
Learning rate	3×10^{-4}
Warmup iterations	100
Maximum iterations	10 000

Training loss analysis. We start from a vocabulary with 469 tokens. At initialization, the training loss corresponds to a uniform distribution over the vocabulary, with an expected entropy of $-\log\left(\frac{1}{469}\right) = \log(469) \approx 6.15$. As Figure S1 shows, the loss decreases steadily during pretraining on ZINC20 and reaches a stable regime around iteration 2,000, converging to a loss close to 0.5.

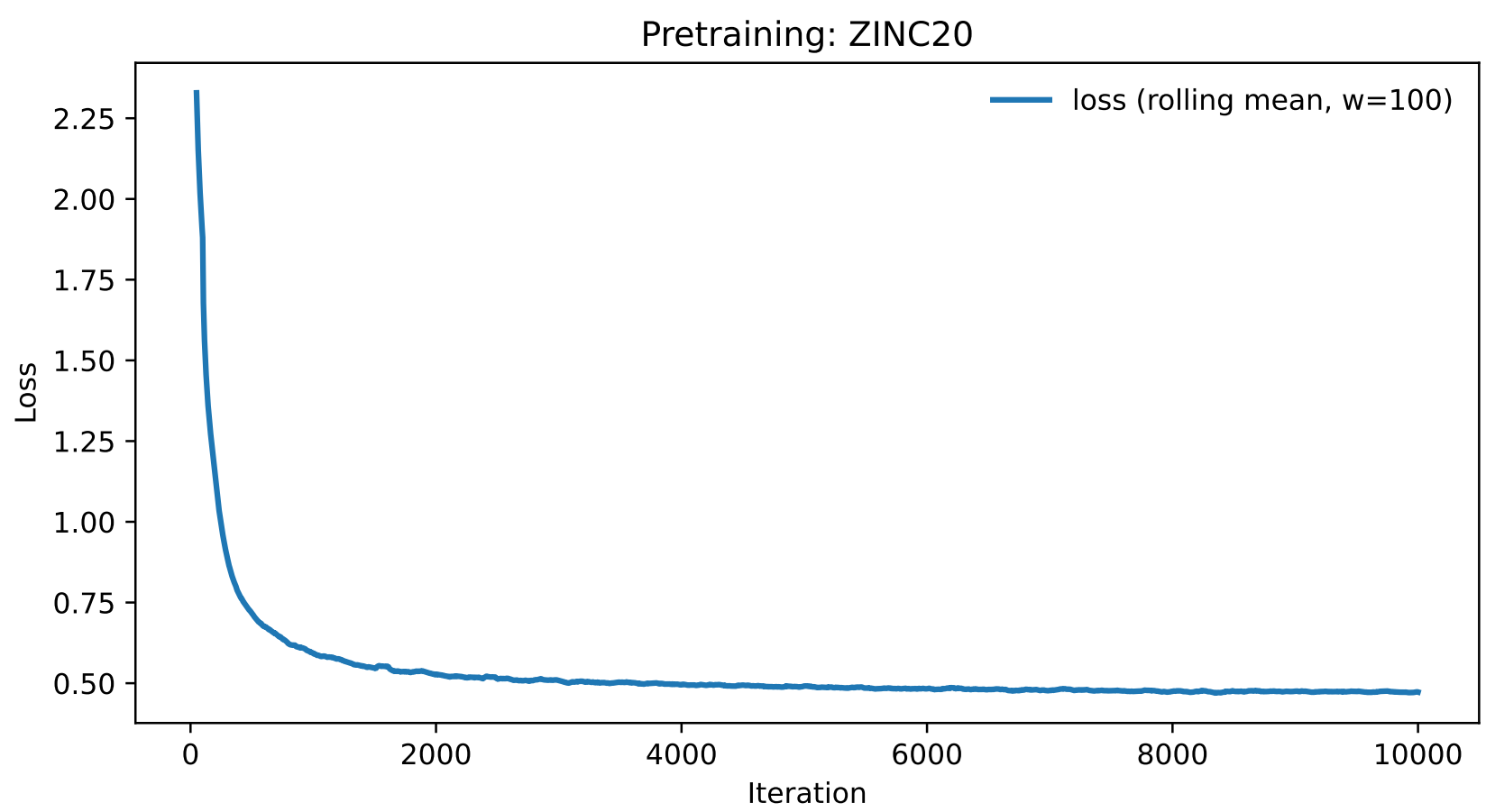

Figure S1: Training loss during pretraining on ZINC20.

Figure S2 shows the training loss during fine-tuning on ChEMBL. The model starts from a lower initial loss, comparing with Figure S1, reflecting that it already learnt general chemical syntax and structure during pretraining. Throughout the 1000 fine-tuning iterations, the loss continues to decrease and eventually stabilizes, although occasional spikes are observed. This behavior indicates adaptation of the learned prior to molecular patterns and tokens that were underrepresented or absent during pretraining.

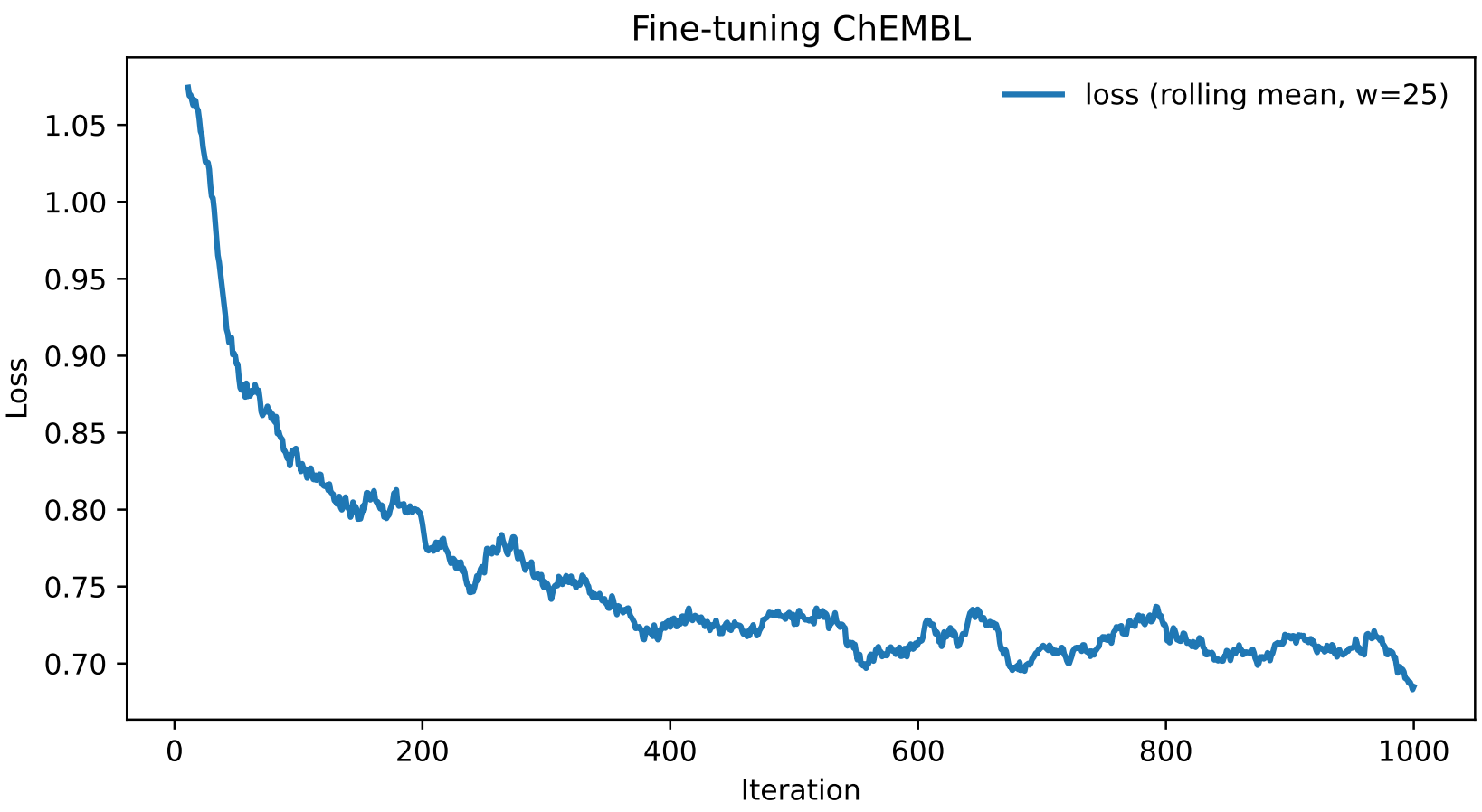

Figure S2: Training loss during fine-tuning on ChEMBL.

During conditional fine-tuning on CrossDocked/Jglaser, the initial loss is substantially higher than in the previous fine-tuning stage (Figure S3). This increase is expected as the input representation is modified to include pocket-related information and amino acid tokens that the model must learn to integrate with the molecular sequence. Despite this distributional shift, training remains stable and converges to a steady loss value.

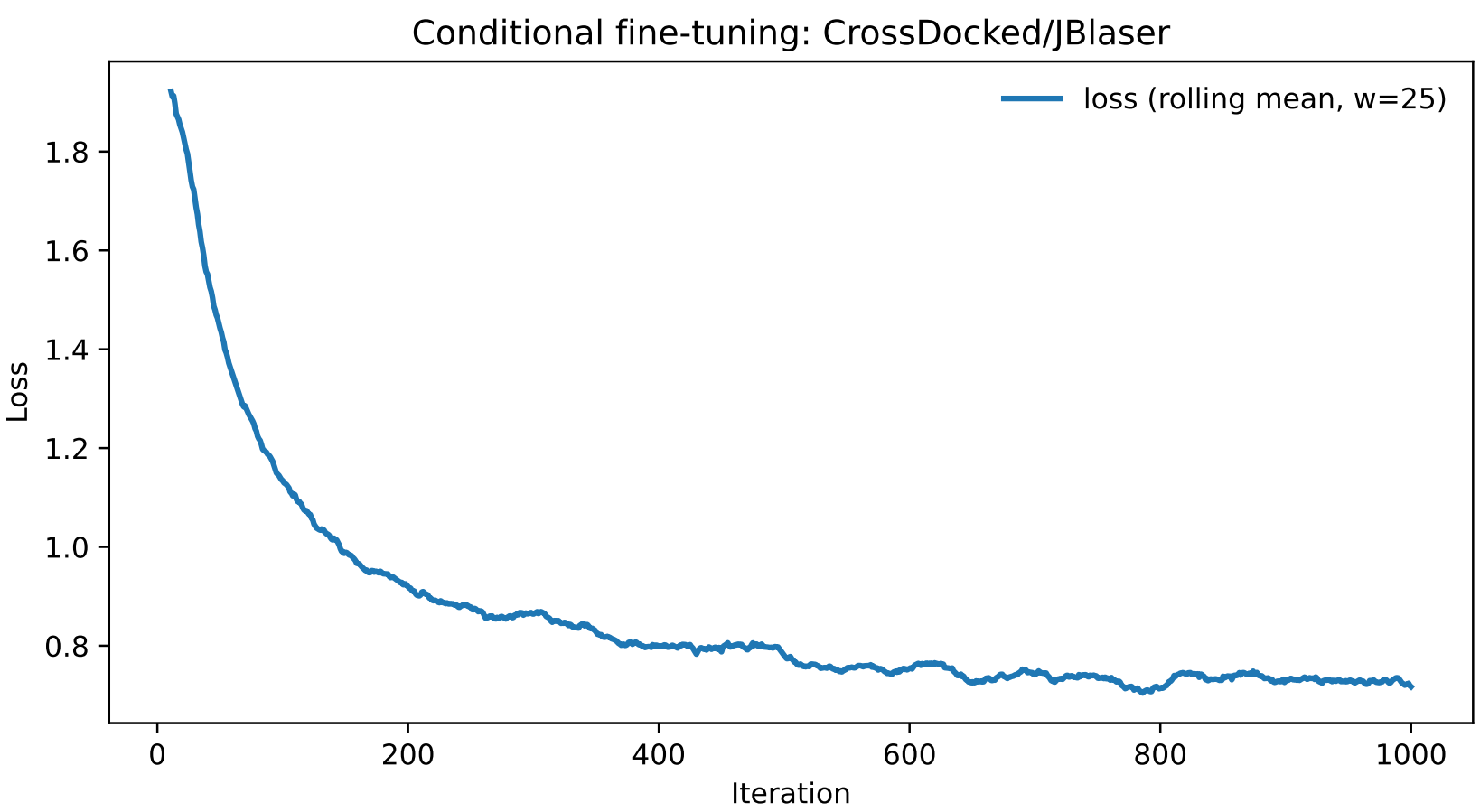

Figure S3: Training loss during conditional fine-tuning on CrossDocked/Jglaser.

Computational cost. All training stages were executed on four NVIDIA Tesla V100 GPUs with 16 GB of memory each. Table S3 summarizes the elapsed training time for each stage.

Table S3: Computational cost of the training pipeline.

**Training stage**	**Time (h)**
Pretraining (ZINC20)	40.85
Fine-tuning (ChEMBL)	1.61
Conditional fine-tuning (CrossDocked/Jglaser)	2.06
**Total**	**44.52**

S3 Reward analysis

To select the final docking reward, we first evaluated the effect of the reward exponent γ_{reward} on reward sensitivity. As Figure S4 shows, $\gamma_{\text{reward}} = 1.3$ provided a balanced response: it increased the reward separation between weak and strong docking scores without making the function excessively sharp. This value was therefore selected to favor molecules with improved docking scores while avoiding overly aggressive optimization.

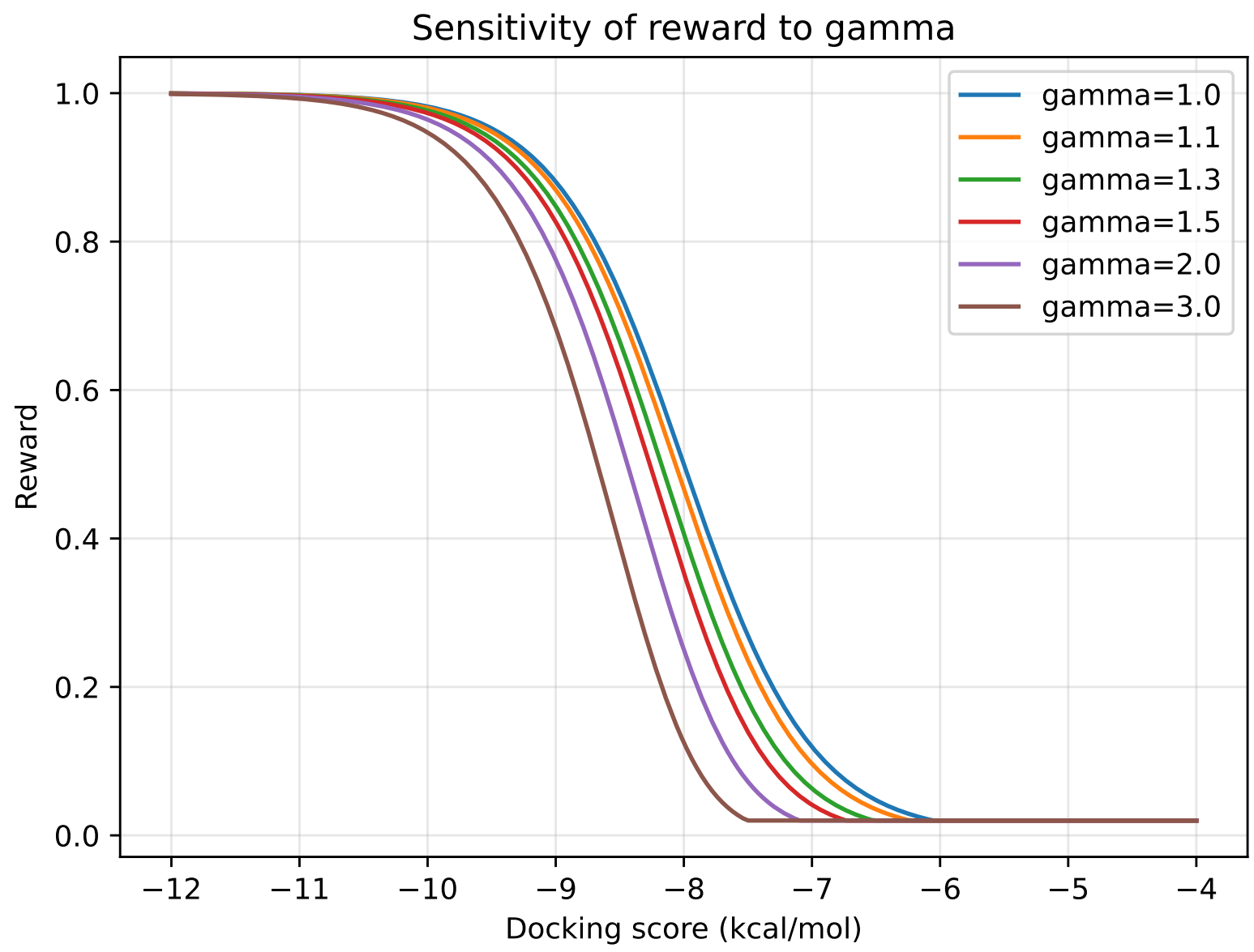

Figure S4: Sensitivity of the docking reward to different exponent values γ_{reward}.

We also compared multiplicative and additive reward formulations combining docking score and logP. As shown in Figure S5, the multiplicative formulation acts as a physicochemical gate: high reward is obtained only when both docking and logP are favorable. In contrast, the additive formulation can still assign relatively high reward when only one of the components is favorable. For this reason, the multiplicative reward was selected, as it better enforces simultaneous optimization of predicted affinity and molecular viability.

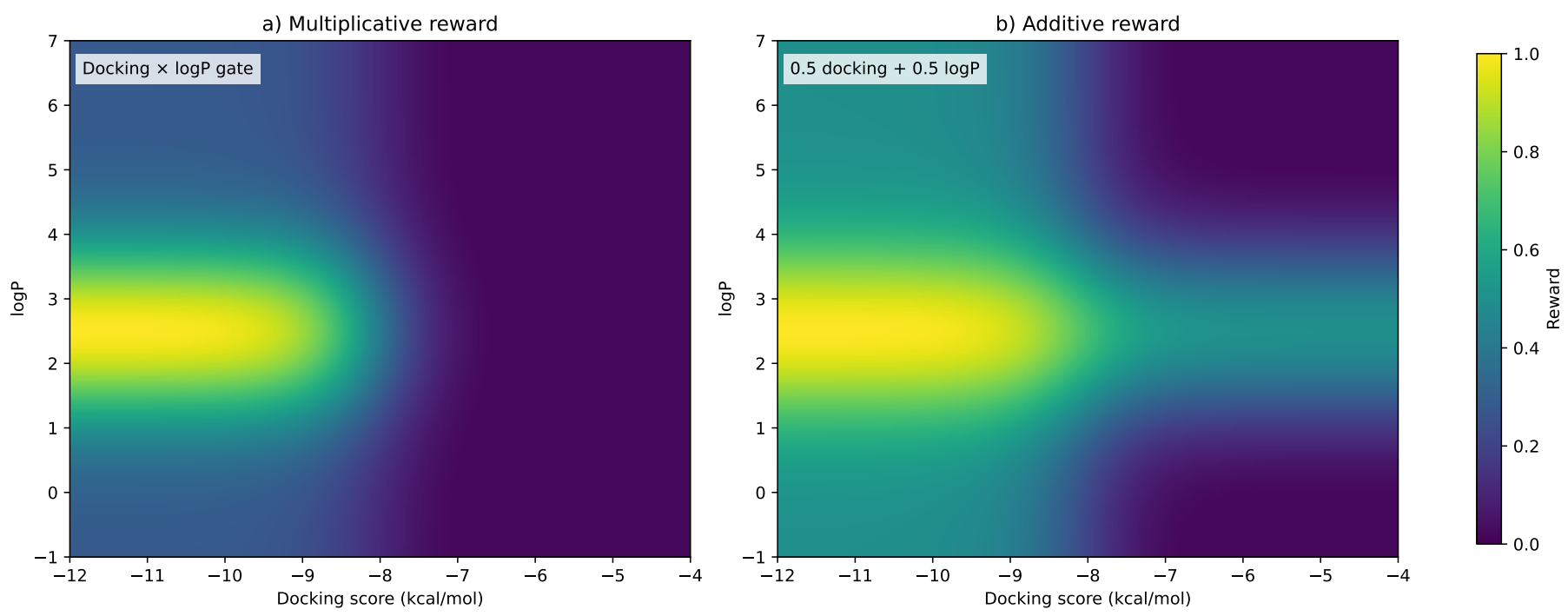

Figure S5: Comparison between multiplicative and additive reward formulations combining docking score and logP.

S4 RL analysis

RL hyperparameters. Table S4 reports the hyperparameters used for PPO-based RL, including rollout configuration, optimization settings, and sampling parameters for sequence generation. Some hyperparameters depend on the conditioning setup. In particular, the prompt size varies depending on whether pocket information is included, and the maximum number of generated tokens is adjusted accordingly to maintain a fixed total sequence length.

Table S4: PPO hyperparameters used.

**Hyperparameter**	**Value**
Batch size (mini-batch)	64
Learning rate	3×10^{-5}
Number of RL epochs	30
Number of rollouts per epoch	256
PPO epochs per update	4
Prompt batch size	256
Sequence length	156
Sampling temperature	0.9
Top-k sampling	10
KL divergence coefficient (β)	0.005
Discount factor (γ)	0.99
Generalized advantage estimation parameter (λ)	0.95
Policy clip range	0.2
Value clip range	0.1
Value function coefficient (λ_{VF})	1.0

PPO losses. We optimize the policy using PPO with a clipped surrogate objective and a clipped value-function regression term. The overall loss is defined as

$$\mathcal{L} = \mathcal{L}_{\mathrm{PG}} + \lambda_{\mathrm{VF}}\,\mathcal{L}_{\mathrm{VF}}, \tag{1}$$

where $\mathcal{L}_{\mathrm{PG}}$ is the policy-gradient surrogate loss with ratio clipping and $\mathcal{L}_{\mathrm{VF}}$ is the value-function loss computed as a clipped mean-squared error between predicted values and generalized advantage estimation returns.

Figure S6 shows the evolution of the three losses. The value-function term $\mathcal{L}_{\mathrm{VF}}$ decreases rapidly and approaches values close to zero. In contrast, the policy-gradient term $\mathcal{L}_{\mathrm{PG}}$ exhibits noticeable oscillations throughout training. This behavior is expected in our setting, as policy updates are driven by high-variance terminal rewards and constrained by PPO clipping.

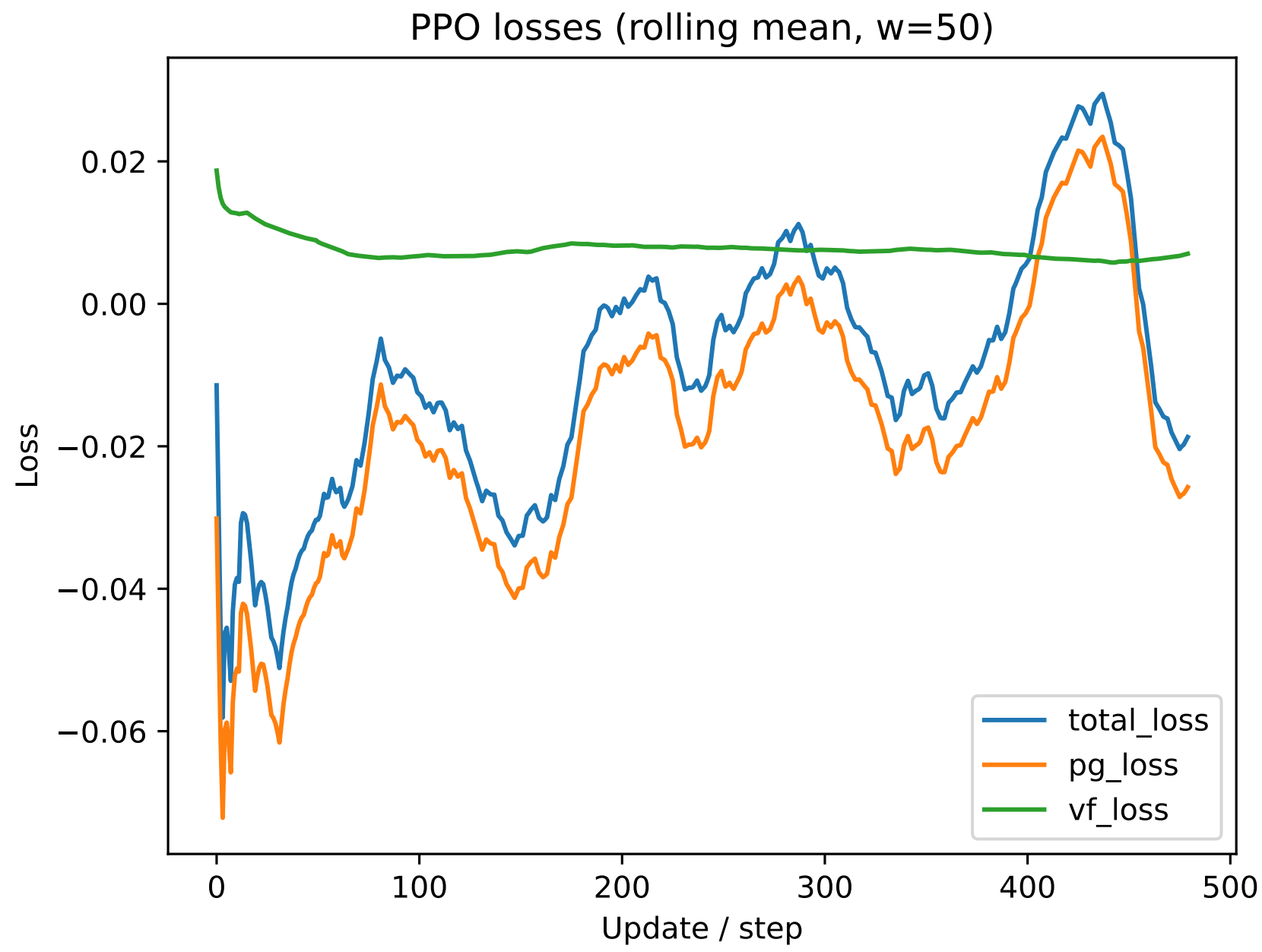

Figure S6: PPO loss components during one experiment.

Reward evolution. Figure S7 shows the average reward per epoch during RL in one optimization experiment. The reward increases during training, indicating that the agent improves its policy with respect to the docking-based reward.

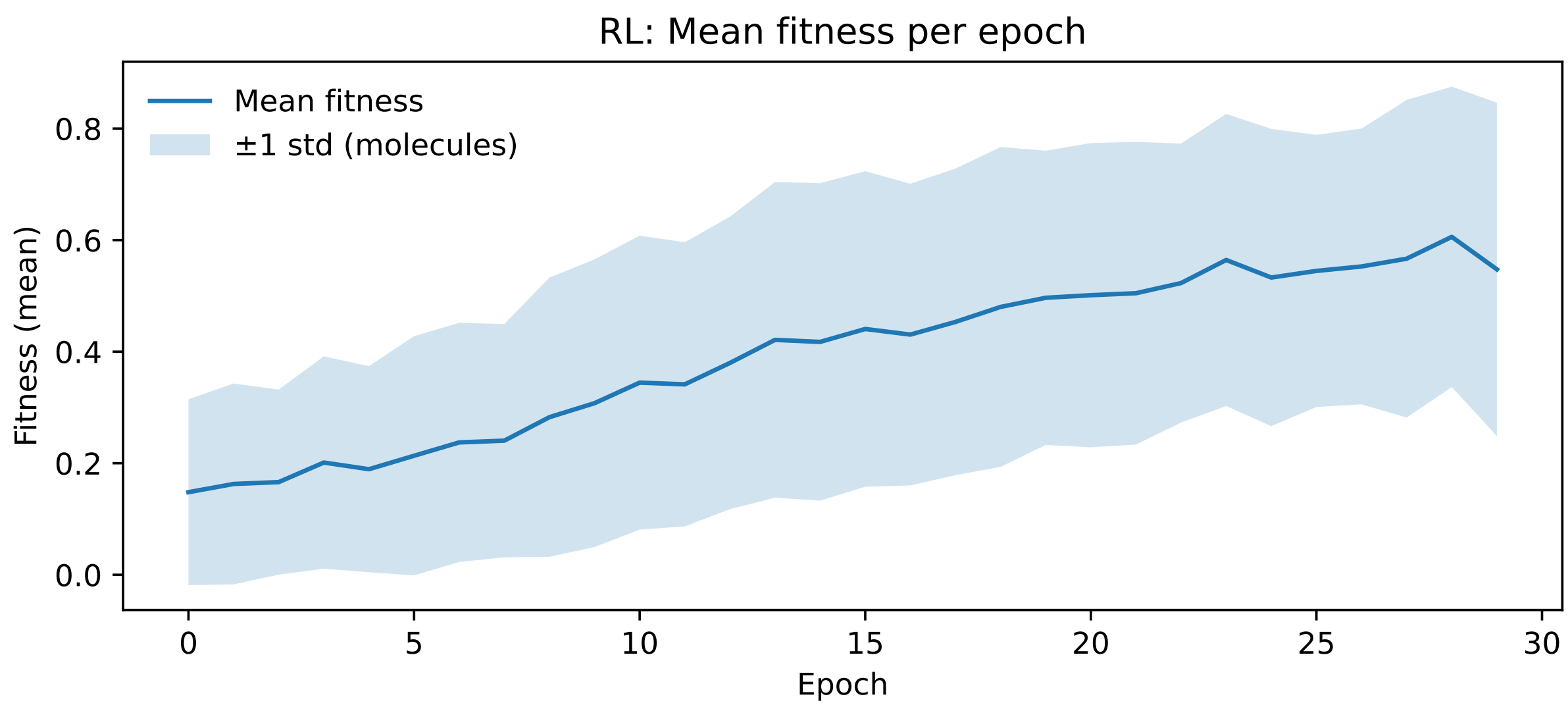

Figure S7: Average reward per epoch during one RL experiment.

RL computational cost. RL is computationally demanding due to the inclusion of the docking-based reward evaluation. In this stage, we used two NVIDIA Tesla V100 GPUs with 16 GB of memory each. On average, a single RL epoch required approximately 25 minutes, dominated by docking evaluations. Consequently, completing 30 RL epochs required approximately 12.5 hours.

S5 Types of conditioning strategies

We compared the ligand-only MolOrgGPT baseline with three pocket-conditioned variants: sequence conditioning, sequence conditioning augmented with ProtT5 residue embeddings, and cross-attention over ProtT5 pocket embeddings.

Pre-RL. Figure S8 shows the docking-score distributions before RL across DYRK1A, BuChE, and BACE-1. Pocket-conditioned models generally produced more favorable docking scores than the ligand-only baseline, indicating that pocket-aware fine-tuning shifted generation toward target-relevant chemical space.

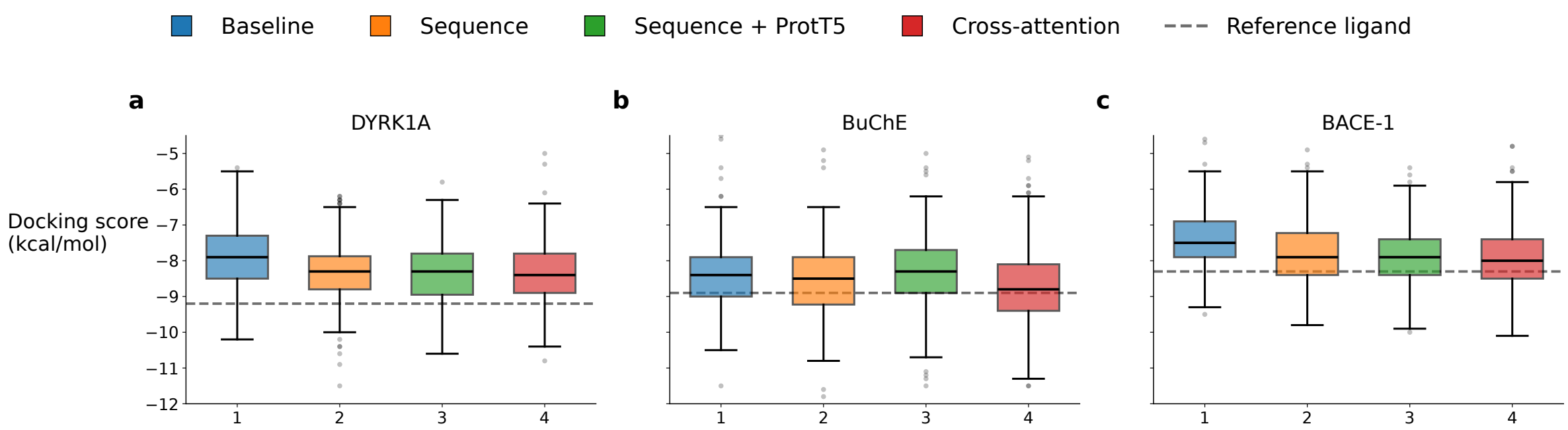

Figure S8: Pre-RL docking-score distributions for the baseline and pocket-conditioned models across three targets: (a) DYRK1A, (b) BuChE, and (c) BACE-1. Dashed lines indicate crystallographic ligand docking scores.

After RL. Figure S9 shows the best-performing epoch after docking-guided RL. RL improved docking performance across all variants, with stronger gains for pocket-conditioned models. The sequence + ProtT5 strategy achieved the best overall behavior, including the lowest median docking scores for DYRK1A and BACE-1 and the highest average fitness, supporting its selection for the final model.

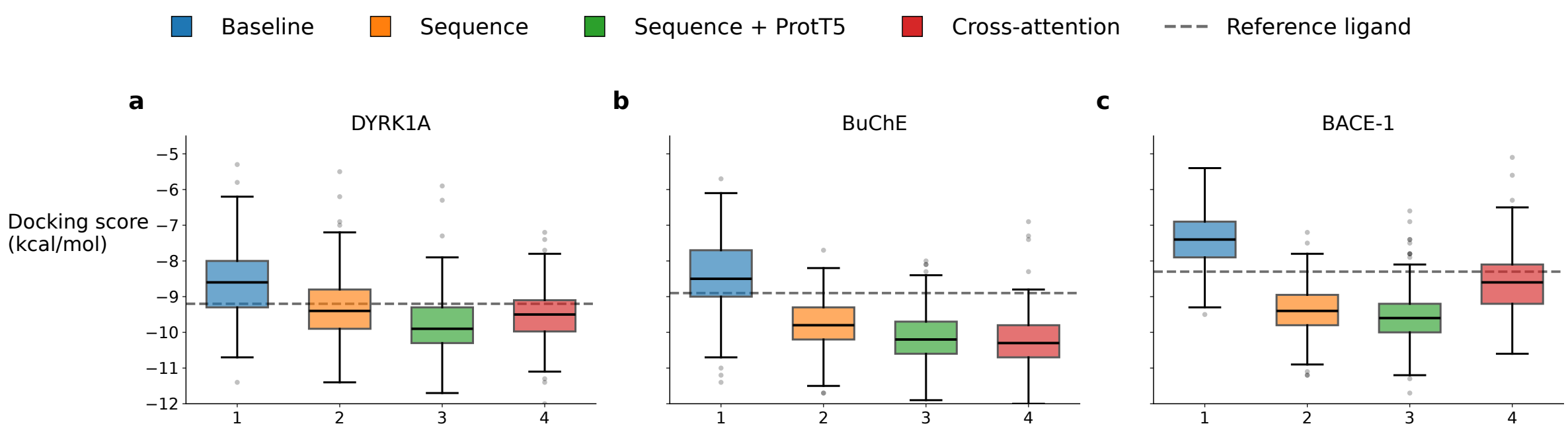

Figure S9: Post-RL docking-score distributions at the best-performing epoch for the baseline and pocket-conditioned models across three targets: (a) DYRK1A, (b) BuChE, and (c) BACE-1. Dashed lines indicate crystallographic ligand docking scores.

Attention–affinity relationship. As Figure S10 shows, the correlation between attention to amino acid tokens and docking performance reached values above 0.2 after the pre-RL stage, suggesting that the model learned pocket–ligand associations during pocket-conditioned fine-tuning. During RL, this correlation progressively decreased because each RL run was optimized against a fixed target pocket. In this setting, the pocket context is constant across generated samples and therefore does not provide a discriminative signal for ranking molecules within the same run. The policy can consequently improve the docking-based reward by modifying ligand

chemical patterns without increasing attention to pocket tokens. Thus, the decay in attention–affinity correlation should be interpreted as a limitation of this attention-based diagnostic under fixed-pocket RL, rather than as direct evidence that pocket conditioning is lost.

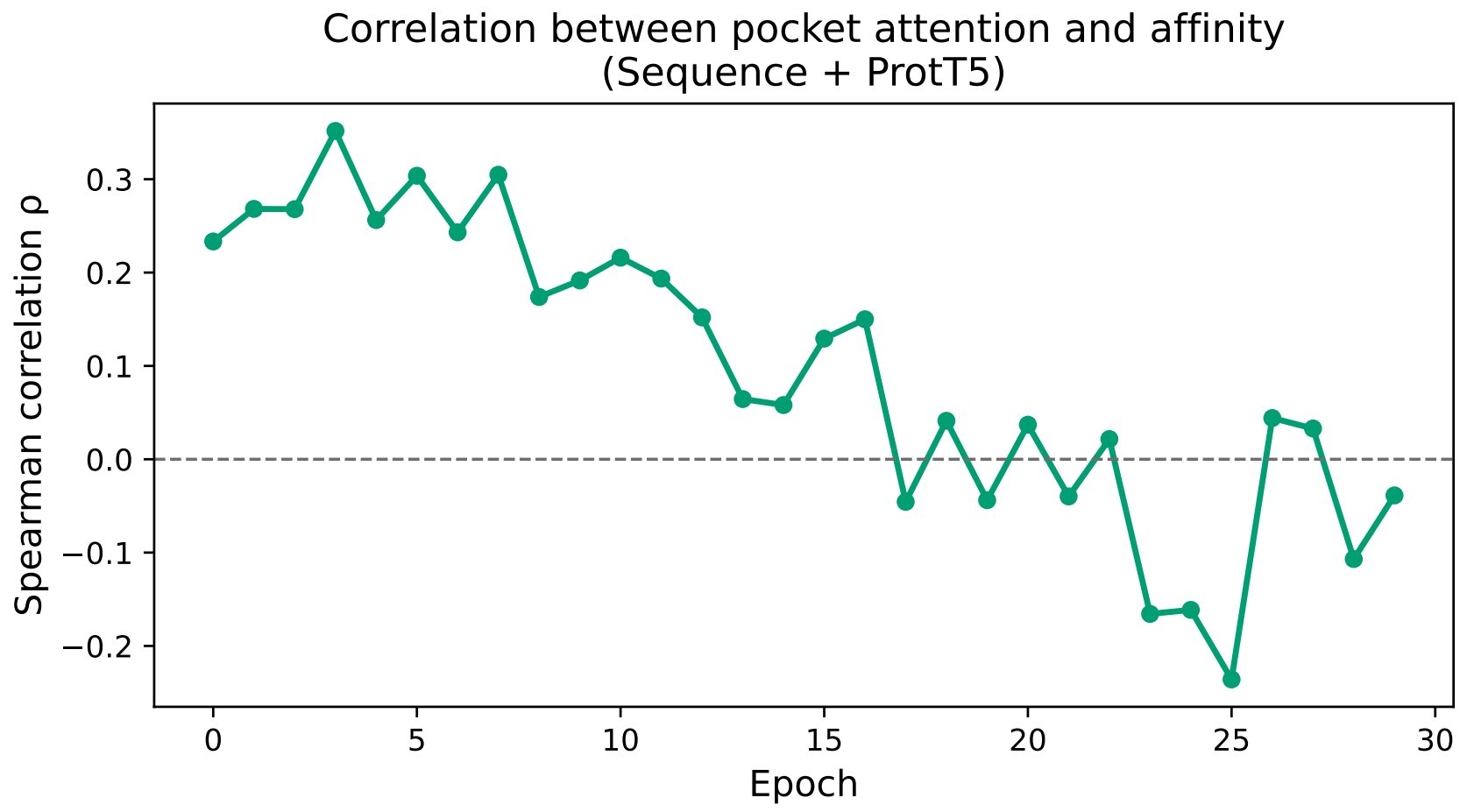

Figure S10: Spearman correlation between attention to amino acid tokens and docking performance during RL.

S6 Docking formula analysis

The AutoDock Vina scoring function decomposes the estimated binding free energy into a weighted sum of physically motivated interaction terms:

$$\begin{aligned} E_{\text{Vina}} = & -0.0356\,\text{Gauss1} - 0.0052\,\text{Gauss2} + 0.8402\,\text{Repulsion} \\ & -0.0351\,\text{Hydrophobic} - 0.5874\,\text{Hbonding} + 0.0585\,N_{\text{rot}}, \end{aligned} \tag{2}$$

where the Gaussian terms model steric complementarity, the repulsion term penalizes atomic clashes, the hydrophobic term rewards nonpolar contacts, the hydrogen-bonding term captures directional polar interactions, and the N_{rot} term penalizes molecular flexibility through the number of rotatable bonds.

Figure S11 reports Pearson correlations between docking scores and intrinsic physicochemical properties of the generated molecules. More favorable docking scores, i.e., more negative values, are associated with increased hydrophobicity and larger molecular size. These properties can negatively affect drug-likeness, inducing an indirect trade-off between docking affinity and overall molecular quality. In particular, while the direct correlation between docking score and QED is weak, increases in logP and MW correlate with reduced QED and higher synthetic accessibility scores, highlighting the multi-objective nature of affinity-driven molecular optimization.

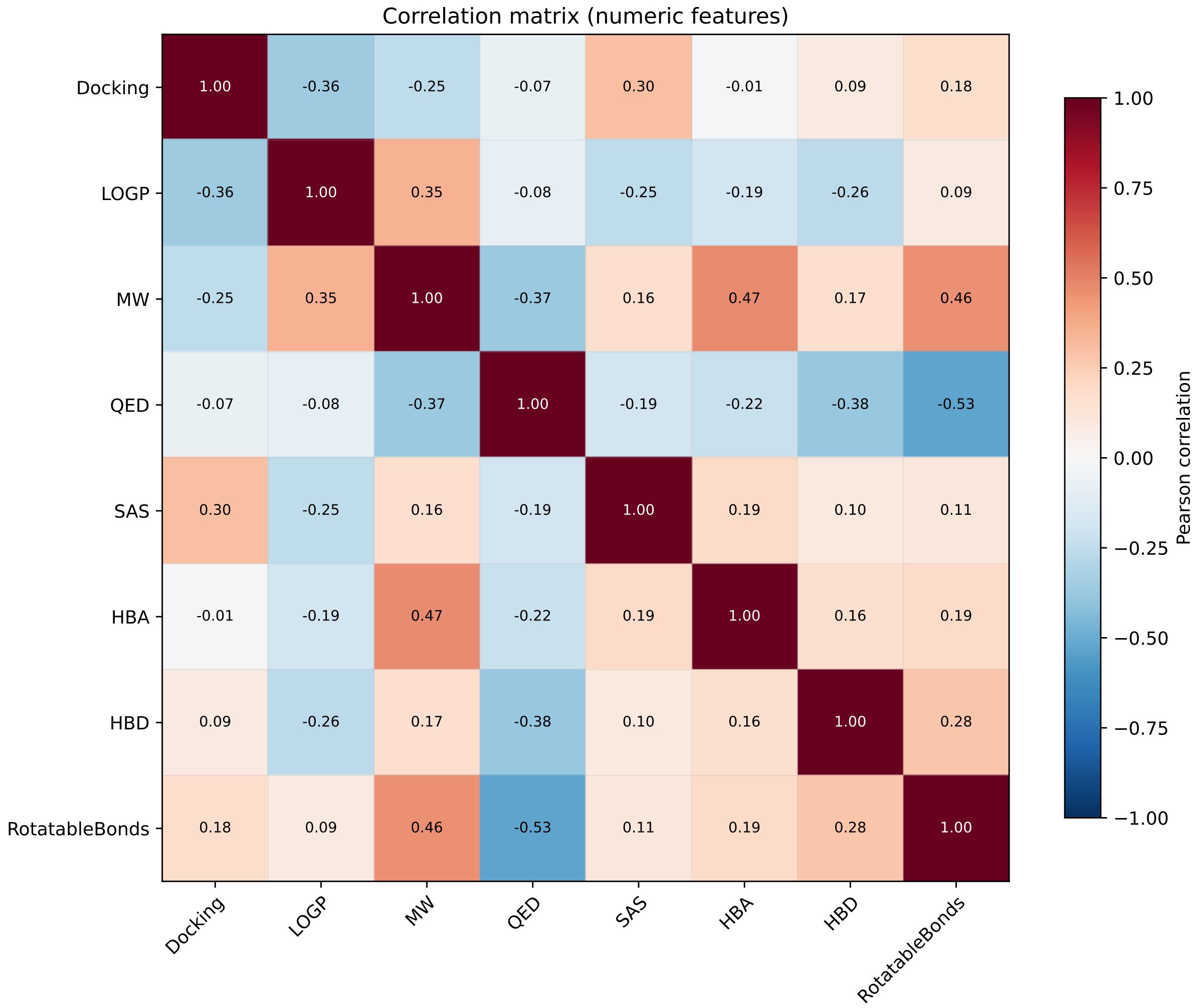

Figure S11: Correlations between docking scores and intrinsic physicochemical properties.

S7 Pocket-level benchmark analysis

Figure S12 compares median docking scores and the percentage of generated molecules passing the physicochemical filters across the 10 protein pockets. PockLigGPT achieved the best docking score in 2 out of 10 pockets and the highest percentage of molecules passing the filters in 9 out of 10 pockets. These results indicate that some baselines obtained stronger docking scores in specific pockets, but often at the expense of poorer physicochemical profiles. Overall, PockLigGPT provided a better balance between predicted affinity and molecular viability.

$\mathbf{N_{pass}}$ denotes the percentage of generated molecules satisfying all physicochemical filters: MW $\in [180, 480]$ Da, logP $\in [0, 5]$, QED > 0.5, SAS < 5, hydrogen bond donors ≤ 5, and hydrogen bond acceptors ≤ 10.

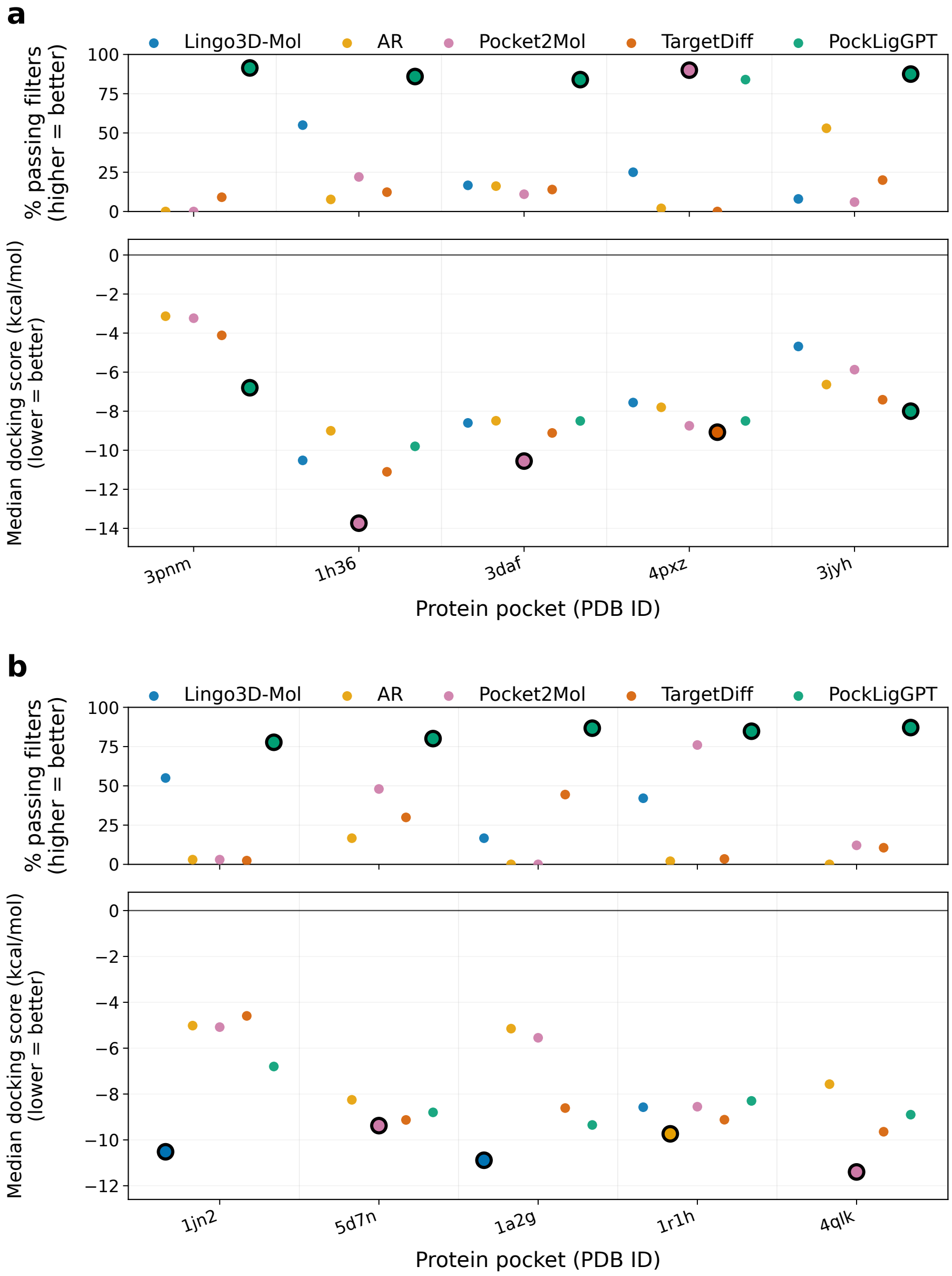

Figure S12: Pocket-level comparison of molecular viability, measured as $\mathbf{N_{pass}}$, and median docking scores across the 10 protein pockets. Lower docking scores indicate stronger predicted binding. Best-performing models per pocket are highlighted.

S8 Top-ranked molecules and docking analysis

The complete list of top-ranked molecules for DYRK1A, BuChE, and BACE-1 is provided in Supplementary Data Table S5. For each molecule, the table reports the SMILES representation, the Glide docking score, and the protein–ligand interactions.

S9 Mathematical formulation of pocket-sequence conditioning

Let

$$\mathcal{P} = (r_1, r_2, \dots, r_L) \tag{3}$$

denote the binding pocket, where r_i is the i-th amino acid residue and L is the number of residues in the pocket.

Discrete pocket conditioning. The pocket sequence is included as a prefix before the ligand tokens:

$$\mathbf{s} = [\texttt{<SOS>}, \texttt{<POCKET>}, r_1, \ldots, r_L, \texttt{</POCKET>}, \texttt{<LIGAND>}, x_1, \ldots, x_k], \tag{4}$$

where $\mathbf{s}$ is the tokenized input sequence, `<SOS>` marks the beginning of the sequence, `<POCKET>` and `</POCKET>` delimit the pocket, and `<LIGAND>` marks the beginning of the ligand representation. The symbols $x_1, \ldots, x_k$ denote the first k SELFIES tokens provided to initialize ligand generation.

Because the model uses causal self-attention, each generated ligand token can attend to all preceding pocket residues and previously generated ligand tokens.

Continuous pocket conditioning. Each pocket residue is also represented by a continuous embedding obtained from a frozen ProtT5 encoder. Let

$$\mathbf{E}^{\text{prot}} = [\mathbf{z}_1, \mathbf{z}_2, \ldots, \mathbf{z}_L]^\top \in \mathbb{R}^{L \times d}, \tag{5}$$

where $\mathbf{z}_i \in \mathbb{R}^d$ is the ProtT5 embedding of residue r_i, and d is the hidden dimension of the GPT model.

These residue embeddings are placed in a tensor

$$\mathbf{P} \in \mathbb{R}^{T \times d}, \tag{6}$$

where T is the length of the complete token sequence. Let s_0 denote the position of the first pocket residue in $\mathbf{s}$. The vector at position t is defined as

$$\mathbf{P}_t = \begin{cases} \mathbf{z}_{t-s_0+1}, & s_0 \leq t < s_0 + L, \\ \mathbf{0}, & \text{otherwise}, \end{cases} \tag{7}$$

where $\mathbf{P}_t \in \mathbb{R}^d$ is the continuous conditioning vector at sequence position t. Therefore, the ProtT5 embeddings are aligned with the corresponding amino-acid tokens, while the remaining positions are filled with zero-padded vectors.

Combined input representation. Let $\mathbf{W}_{\text{tok}}$ denote the token-embedding matrix and $\mathbf{W}_{\text{pos}}$ the positional-embedding matrix. At position t, the transformer input is

$$\mathbf{h}_t^{(0)} = \text{Dropout}\left(\mathbf{W}_{\text{tok}}[s_t] + \mathbf{W}_{\text{pos}}[t] + \text{LN}_f(\mathbf{P}_t)\right), \tag{8}$$

where s_t is the token located at position t, $\mathbf{W}_{\text{tok}}[s_t] \in \mathbb{R}^d$ is its token embedding, $\mathbf{W}_{\text{pos}}[t] \in \mathbb{R}^d$ is its positional embedding, and LN_f is the layer-normalization operation applied to the continuous pocket representation before addition.

Thus, the model receives pocket information through two complementary channels: the amino-acid identities are included as discrete tokens, while the ProtT5 representations provide continuous residue-level information. Both signals are combined before the sequence is processed by the transformer blocks.
